# Balanced activities of Hsp70 and the ubiquitin proteasome system underlie cellular protein homeostasis

**DOI:** 10.3389/fmolb.2022.1106477

**Published:** 2023-01-04

**Authors:** Areeb Jawed, Chi-Ting Ho, Tomas Grousl, Aseem Shrivastava, Thomas Ruppert, Bernd Bukau, Axel Mogk

**Affiliations:** ^1^ Center for Molecular Biology of Heidelberg University (ZMBH), University of Heidelberg, Heidelberg, Germany; ^2^ German Cancer Research Center (DKFZ), Heidelberg, Germany

**Keywords:** protein quality control, Hsp70, 26S proteasome, chaperone, protein sequestration

## Abstract

To counteract proteotoxic stress and cellular aging, protein quality control (PQC) systems rely on the refolding, degradation and sequestration of misfolded proteins. In *Saccharomyces cerevisiae* the Hsp70 chaperone system plays a central role in protein refolding, while degradation is predominantly executed by the ubiquitin proteasome system (UPS). The sequestrases Hsp42 and Btn2 deposit misfolded proteins in cytosolic and nuclear inclusions, thereby restricting the accessibility of misfolded proteins to Hsp70 and preventing the exhaustion of limited Hsp70 resources. Therefore, in yeast, sequestrase mutants show negative genetic interactions with double mutants lacking the Hsp70 co-chaperone Fes1 and the Hsp104 disaggregase (*fes1Δ hsp104Δ*, *ΔΔ*) and suffering from low Hsp70 capacity. Growth of *ΔΔbtn2Δ* mutants is highly temperature-sensitive and results in proteostasis breakdown at non-permissive temperatures. Here, we probed for the role of the ubiquitin proteasome system in maintaining protein homeostasis in *ΔΔbtn2Δ* cells, which are affected in two major protein quality control branches. We show that *ΔΔbtn2Δ* cells induce expression of diverse stress-related pathways including the ubiquitin proteasome system to counteract the proteostasis defects. Ubiquitin proteasome system dependent degradation of the stringent Hsp70 substrate firefly Luciferase in the mutant cells mirrors such compensatory activities of the protein quality control system. Surprisingly however, the enhanced ubiquitin proteasome system activity does not improve but aggravates the growth defects of *ΔΔbtn2Δ* cells. Reducing ubiquitin proteasome system activity in the mutant by lowering the levels of functional 26S proteasomes improved growth, increased refolding yield of the Luciferase reporter and attenuated global stress responses. Our findings indicate that an imbalance between Hsp70-dependent refolding, sequestration and ubiquitin proteasome system-mediated degradation activities strongly affects protein homeostasis of Hsp70 capacity mutants and contributes to their severe growth phenotypes.

## Introduction

The cellular proteostasis network limits the accumulation of misfolded protein species, preventing aberrant and potentially toxic interactions and ensuring protein homeostasis. The network relies on three main strategies: the refolding of non-native proteins by ATP-dependent chaperones, their removal by proteolytic systems and their sequestration into inert inclusions (Sontag et al., 2017). In *Saccharomyces cerevisiae* the ATP-dependent Hsp70 chaperone system represents the central component of the folding branch of the protein quality control system (PQC). The alleles *ssa1* to *ssa4* encode for the Hsp70 proteins and their simultaneous deletion is lethal, underlining their crucial relevance for PQC. Hsp70 is supported by co-chaperones that either assist in coupling ATP hydrolysis with substrate binding (J-domain proteins: e.g. Ydj1, Sis1, Apj1) or accelerate nucleotide exchange (NEFs: e.g. Sse1/2, Fes1) (Rosenzweig et al., 2019). Hsp70 additionally cooperates with the AAA + family member Hsp104 in protein disaggregation and the Hsp90 chaperone in folding of specific protein clients (Rosenzweig et al., 2019). A major route to degradation of misfolded protein is the ubiquitin proteasome system (UPS) in yeast cells. The system relies on the ubiquitination of misfolded proteins by specific E3 ligases (San1, Ubr1, Rsp5) and the subsequent degradation by the 26S proteasome (Finley and Prado, 2020). In case of dysfunctional proteasomes a specific degradation pathway termed proteophagy has been identified. This system involves the removal of ubiquitinated proteasomes by autophagy (Peters et al., 2015; Marshall et al., 2016). The sequestration of misfolded proteins represents the most recently described PQC strategy (Kaganovich et al., 2008). In yeast, the small heat shock protein (sHsp) Hsp42 and the heat shock protein Btn2 target misfolded proteins to cytosolic (CytoQ, Q-bodies) and nuclear (INQ) inclusions (Specht et al., 2011; Escusa-Toret et al., 2013; Miller et al., 2015).

How the diverse branches of PQC cooperate is an important yet largely unresolved question. Do they function as compensatory backup systems in case one branch is dysfunctional or overwhelmed? Or do they rather compete for substrate binding and triage substrates towards different fates? In the latter case the activities of the different branches need to be tightly balanced to ensure substrate targeting to correct pathways. Information on the interaction between refolding and proteolytic pathways is scarce and inconsistent in parts. Positive genetic interactions between yeast *ssa1Δssa2Δ* mutants and UPS activity support a competition model: the temperature-sensitive growth of the Hsp70 mutant could be partially rescued by reducing ubiquitin-dependent degradation *via* the 26S proteasome (Baxter and Craig, 1998; Oling et al., 2014). This implies that mistargeting of Hsp70 substrates towards proteolytic pathways contribute to *ssa1Δssa2Δ* growth defects. However, reducing ubiquitin-dependent protein degradation by overproduction of the deubiquitinase Ubp3 can have diverse consequences on substrate stabilities in Hsp70-deficient cells, pointing to a more complex relationship (Oling et al., 2014). Notably, the expression of both, refolding chaperones and the UPS system, is upregulated upon heat shock (Muhlhofer et al., 2019). This is explained by Hsf1-driven expression of Rpn4, the central transcription factor controlling transcription of UPS components (Hahn et al., 2006). This intertwined co-regulation of both PQC branches rather speaks for cooperation between both activities.

The interplay between the sequestrases Hsp42 and Btn2 and the refolding and degrading branches of the PQC system was only recently analyzed. Negative genetic interactions between sequestrase mutants and temperature-sensitive Hsp70 capacity mutants suggest that sequestrases restrict substrate accessibility to prevent Hsp70 overload (Ho et al., 2019). This link is also supported through direct interactions between Btn2 and the Hsp70 co-chaperone Sis1 and the cooperation between Hsp42 and the Hsp70 system in substrate refolding (Ho et al., 2019). While no genetic interactions were observed between the sequestrases and the UPS system (Ho et al., 2019), substrates sequestered *via* Btn2 at INQ were also shown to be targeted for proteasomal degradation (den Brave et al., 2020). This points to substrate-specific effects leaving the impact of cellular inclusions on substrate fate and refolding vs degrading activities open.

Here, we dissected the interplay between the three major proteostasis activities by utilizing yeast mutant cells suffering from very low Hsp70 capacity due to deletions of Hsp70 partner chaperones and the nuclear sequestrase Btn2. This allowed us to dissect the role of the UPS system when both other branches of PQC are weakened. We show that reducing UPS activity rescues growth and proteostasis defects of the Hsp70 capacity mutants. Notably, the UPS is strongly induced in these mutants, creating an imbalance of refolding and proteolytic activities and targeting the Hsp70 substrate Luciferase to degradation. UPS induction thus causes collateral damage and re-balancing of refolding and degrading activities is required to restore protein homeostasis and attenuate triggered stress responses.

## Materials and methods

### Yeast strains, growth conditions and spot tests

All yeast strains used in this study are derived from BY4741 and listed in the Supplementary Table S1. Yeast genome was edited using the homologous recombination technique as described (Janke et al., 2004). Desired genomic modifications were PCR amplified with flanking sequences homologous to the target genomic loci and suitable selection markers. PCR products were transformed in yeast cells and selected for the marker (auxotrophy or drug resistance). Correct integration at the locus was verified by PCR. Yeast cells were grown in liquid YPD or synthetic dropout (SC) media at indicated temperatures. SD medium was supplemented with 2% (w/v) glucose (Glu). Growth plates contained bacto-agar to a final volume of 2% (w/v). For spot tests yeast cells were grown in SC or YPD medium to mid-log phase (OD_600_ = .6–.8) and then diluted to OD_600_ = .2. Cells were 5-fold serially diluted and spotted on YPD or SC agar plates. Plates were incubated at diverse temperatures for two to three days before documentation.

### Preparation of total cell lysates

Yeast cells were grown to .8 OD_600_. 800 μL of cell culture was mixed with 150 μL of ice cold 1.85 M NaOH, vortexed and incubated on ice for 10 min 150 μL of 55% (v/v) TCA was added and the suspension was gently mixed, followed by a further incubation on ice for 10 min. The mixture was centrifuged at 13,000 rpm, 4°C, 15 min. The supernatant was discarded and the pellet was resuspended in 100 μL of HU buffer (8 M Urea, 5% (w/v) SDS, 200 mM Tris pH 6.8, 1 mM EDTA, 1.5% (w/v) DDT, .1% (w/v) bromophenol blue) per 1 OD_600_ equivalent. The resuspended pellet was incubated at 65°C for 10 min before analysis by SDS-PAGE.

### Western blotting

SDS-PAGEs were transferred to PVDF membranes by semi-dry blotting. Membranes were subsequently blocked with 3% BSA (w/v) in TBS-T. Antibodies and used dilutions are listed in Supplementary Table S2. Anti-rabbit alkaline phosphatase conjugate (Vector Laboratories) was used as secondary antibody (1:20.000). Blots were developed using ECF™ Substrate (GE Healthcare) as reagent and imaged *via* Image-Reader LAS-4000 (Fujifilm). Western blotting was performed in two or more independent experiments each and representative results are provided.

### Microscopy and image processing

For live cell imaging yeast cells were harvested by centrifugation, resuspended in SC medium and directly applied onto microscope slides. To collect cell images, optical sections of .2 µm were acquired to image the whole cell volume using a widefield system (xcellence IX81, Olympus) equipped with a Plan-Apochromat 100x/NA 1.45 oil immersion objective and an EMCCD camera (Hamamtsu). Acquired z-stacks were deconvolved with xCellence software (Olympus) using the Wiener Filter. All further processing and analysis of digital images was performed with ImageJ (NIH).

### Luciferase assay

Yeast cells expressing GFP-NLS-LuciDM were grown in SC-Leucine media overnight at 25°C. Cells were diluted to OD_600_ .2 and incubated at 25°C, 28°C or 30°C until they reached mid-log phase (OD_600_ .6–.8). Luciferase activities were subsequently determined using a Lumat LB 9507 luminometer (Berthold Technologies). Luciferase activities were normalized to the same OD_600_ value and the relative activity determined in WT cells was set to 100%.

### Fluorescence activated cell sorting analysis

To monitor Hsf1-dependent gene expression GFP was placed under control of the *btn2* promoter. To increase reporter sensitivity, the unstable N-terminal domain of Btn2 was fused C-terminally to GFP. GFP fluorescence (excitation: 488 nm, emission: 530 nm) was determined by FACS (Fluorescence Activated Cell Sorting) using the BD FACS Canto machine. Cultures of yeast strains were grown at 25°C or 30°C until log phase (.6–.8 OD_600_). 200 µL of cell culture were added to a flat-bottom 96-well plate with four technical replicates per strain. FACS measured GFP fluorescence of 10.000 cells per strain. The GFP values were normalized by subtracting the values of a negative control.

### Statistical analysis

Unpaired t tests were conducted using GraphPad Prism six software. Data distribution was assumed to be normal.

### SILAC, mass spectrometry and data analysis

Yeast arg*4*∆ *lys1*∆ cells were grown in SC medium in presence of heavy isotopes of l-arginine and l-lysine (.02 mg/ml Arg4 and .03 mg/ml Lys8), while respective derivatives of proteostasis mutants (*fes1*∆ *hsp104*∆ (*∆∆*), *∆∆btn2∆*, *∆∆btn2∆rpn4∆*) were grown in presence of regular (light) amino acids. Total protein extracts were prepared and equal amounts of WT (K8/R10) and mutant total extracts (each .1 OD_600_) were mixed and separated by SDS-PAGE for about 2 cm. The SDS gel was stained for 4 h in Quick-Coomassie (Abcam), followed by destaining in sterile water overnight. Each lane was cut into four pieces and processed as described previously (Barenz et al., 2013). In brief, proteins in gel pieces were reduced, alkylated and digested with trypsin. Peptides were extracted from the gel pieces, concentrated in a vacuum centrifuge and dissolved in 15 µL .1% TFA. Nanoflow LC-MS2 analysis was performed with an Ultimate 3,000 liquid chromatography system coupled to an QExactive HF (Thermo Fisher). An in-house packed analytical column (75 μm × 200 mm, 1.9 µm ReprosilPur-AQ 120 C18 material (Dr. Maisch, Germany) was used. Peptides were separated in a 120 min linear gradient (3%–40% B) (Solvent A: .1% formic acid/1% acetonitrile, solvent B: .1% formic acid, 89.9% acetonitrile). The mass spectrometer was operated in data-dependent acquisition mode, automatically switching between MS and MS2. MS spectra (m/z 400–1600) were acquired in the Orbitrap at 60,000 (m/z 400) resolution and MS2 spectra were generated for up to 15 precursors with normalized collision energy of 27%. The MS/MS spectra were searched against the Uniprot KB yeast database and a contaminants database (MaxQuant 1.6.2.6) using Proteome Discoverer 2.5 with Sequest (Thermo Fisher Scientific). Trypsin was specified as enzyme. Carbamidomethyl was set as fixed modification of cysteine and oxidation (methionine), deamidation (asparagines, glutamine) and acetylation (protein N-terminus) as variable modifications. Mass tolerance was set to 10ppm and .5 Da for MS and MS/MS, respectively. A false discovery rate was set to .01 using Percolator.

Results were exported and analyzed by Perseus software (1.6.13.0) (Tyanova and Cox, 2018). Heavy/Light ratios of peptides were determined for comparing proteomes of WT and mutant strains. The calculation of ratios of ratios allowed for comparison of mutant strains. Proteins with a peptide count below four were discarded from the analysis. Lists of proteins that were 1.5-fold up- or down-regulated in two independent experiments were generated and used to create volcano plots of mutant strains showing differentially regulated protein against the WT control. Next, the same protein list was uploaded and analyzed by the STRING (v10) software tool to generate protein-protein interaction networks. STRING is an online database and software tool for both known and computationally predicted protein-protein interactions including both direct (physical) and indirect (functional) associations. The network uploaded onto STRING was filtered to include interactions, based on textmining, experimental evidence and existing database. The derived networks were further analyzed by Cytoscape software allowing for their visualization. Cytoscape is an open-source software wherein users can install various applications required for the analysis and visualization of high-throughput expression data. For this study, the MCODE algorithm was used to identify and isolate dense clusters of protein interactions within the entire network. Clusters usually contain proteins working together either in specific response pathways or executing similar functions in the cell. The final visualization of protein clusters was done *via* the yFiles layout algorithm, which is a diagramming program installed onto Cytoscape and is used for arranging and visualizing graph structures. The heat maps of up- and down-regulated proteins were generated by Prism9 software after importing protein identities, their expression changes and their clustering patterns. The YEASTRACT + database (www.yeastract.com), which is an online repository of regulatory associations between transcription factors (TF) and their target genes/proteins, was used to identify the transcription factors regulating the identified protein clusters.

## Results

### Limiting ubiquitin proteasome system activity partially rescues Hsp70 capacity mutants

**FIGURE 1:**
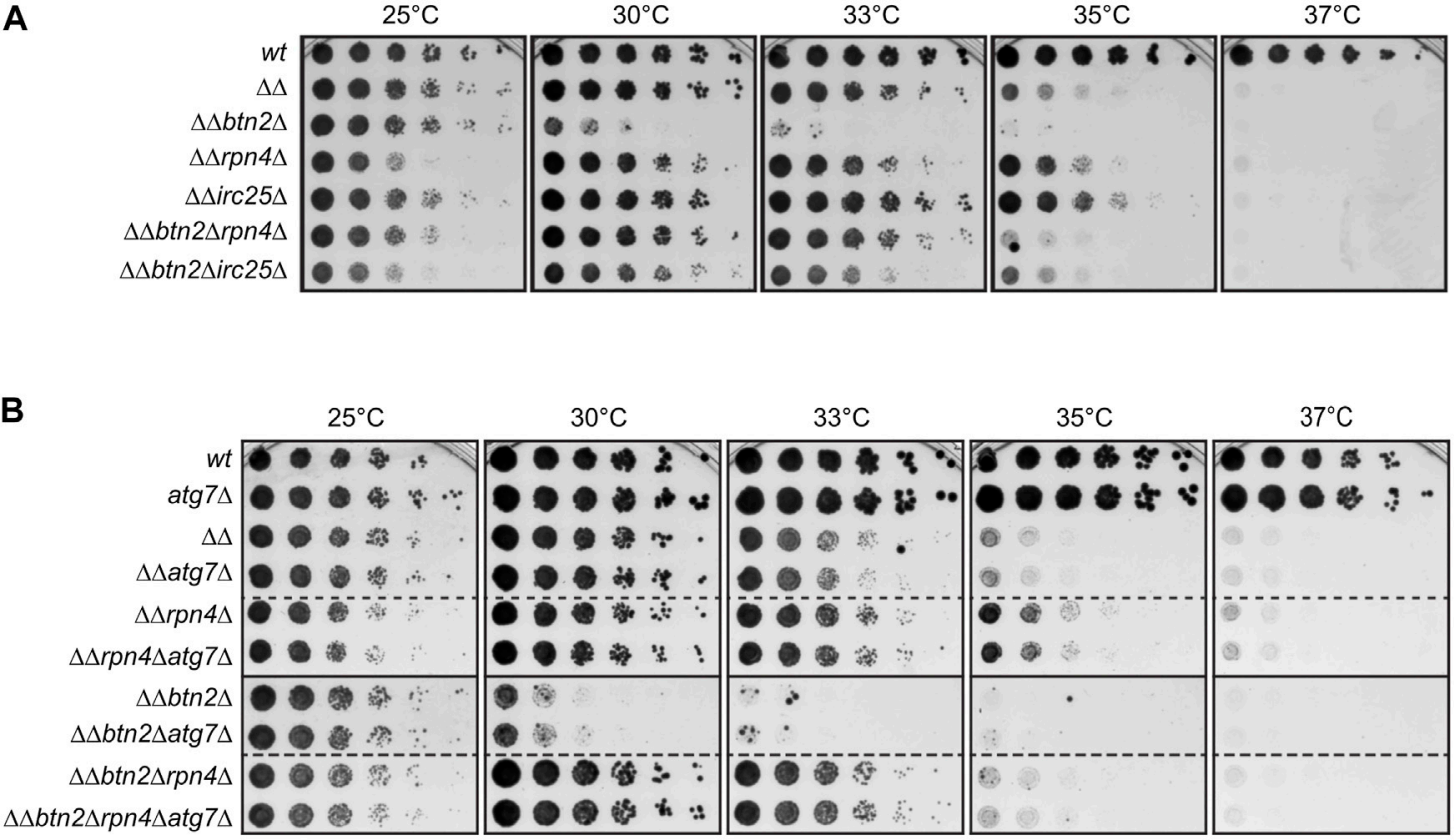
Reducing UPS activity partially rescues growth defects of yeast mutants with low Hsp70 capacity. **(A,B)** Five-fold serial dilutions of indicated *S. cerevisiae* wild type (wt) and proteostasis mutant cells (*ΔΔ: hsp104Δ fes1Δ*) were spotted on YPD-plates and incubated at indicated temperatures for 2 days. Dashed lines indicate that spot tests from different plates are cropped and shown together for comparative analysis.

We previously showed that Btn2 and Hsp42 sequestrases become essential for growth of *S. cerevisiae fes1Δ hsp104Δ* (termed *ΔΔ*) mutant cells. *ΔΔ* cells lack the nucleotide exchange factor (NEF) Fes1 of the Hsp70s Ssa1 to Ssa4 (Kabani et al., 2002) and the AAA + disaggregase Hsp104, which cooperates with Hsp70 in protein disaggregation (Glover and Lindquist, 1998). Both Hsp70 partners displace substrates from Hsp70 and in consequence the working capacity of Hsp70 is low in *ΔΔ* cells, resulting in a temperature-sensitive growth phenotype at ≥35 °C (Figure 1A) (Ho et al., 2019). This phenotype is further aggravated upon additional loss of the Btn2 and Hsp42 sequestrases abrogating growth at 30°C and 33°C, respectively (Ho et al., 2019). We made use of the strong growth defect of *ΔΔbtn2Δ* cells to genetically dissect the interconnection between the UPS and sequestration and Hsp70 refolding activities. To lower UPS activity we either deleted *rpn4*, which controls expression of proteasomal genes, or *irc25*, which encodes for an assembly factor of the 26S proteasome (Mannhaupt et al., 1999; Kusmierczyk et al., 2008). *Δrpn4* and *Δirc25* cells therefore harbor reduced levels of functional 26S proteasomes. Remarkably, this reduction of UPS activity did not enhance the growth defects of *ΔΔbtn2Δ* cells but instead led to a recovery of cellular fitness up to 35C (Figure 1A). A growth rescue was also observed for *ΔΔrpn4Δ and ΔΔirc25Δ* cells, indicating that the effect is linked to limitations in Hsp70 capacity. To substantiate our findings we chemically reduced 26S proteasome activity in *ΔΔ* and *ΔΔbtn2Δ* cells by adding MG132, a proteasome active site inhibitor (Supplementary Figure S1A). We observed a growth rescue of *ΔΔbtn2Δ* at 30°C in presence of MG132, though it was less pronounced as compared to genetically affecting 26S functionality. We also overexpressed Rpn4 from the 2μ plasmid Yep13 as complementary strategy to test for the consequences of increased 26S proteasome levels in *ΔΔ* and *ΔΔbtn2Δ* cells and observed an increased temperature sensitivity of *ΔΔ* cells (Supplementary Figure S1B).

We considered the possibility that growth rescue of cells with low Hsp70 capacity is not directly caused by lowering UPS activity but by an increased activity of the autophagic-lysosomal pathway (ALP). ALP can work as backup system when UPS activity is compromised, allowing for clearance of damaged proteins by this alternative degradation pathway (Lilienbaum, 2013; Houck et al., 2014). We therefore tested whether the growth rescue noticed in *ΔΔrpn4Δ* and *ΔΔbtn2Δrpn4Δ* cells is caused by enhanced ALP activity and deleted *atg7* in the proteostasis mutant cells. Atg7 is essential for ALP activity in yeast cells (Mizushima et al., 1998). We did not observe any genetic interactions between the diverse proteostasis mutants (*ΔΔ, ΔΔbtn2Δ, ΔΔrpn4Δ*, *ΔΔbtn2Δrpn4Δ*) and *atg7Δ* (Figure 1B) as growth remained unaltered upon combining the deletions. This excludes a role of the ALP in the growth rescue mediated by *rpn4* deletion and indicates that *ΔΔ* and *ΔΔbtn2Δ* cells suffer directly from an imbalance in Hsp70 and UPS activities. We focused our further analysis on cells harboring a *rpn4* deletion to avoid additional effects of unassembled proteasomes accumulating upon *irc25* deletion.

### Lowering ubiquitin proteasome system activity prevents exhaustive Hsp70 substrate degradation and proteostasis breakdown

To directly monitor the impact of varying interconnection between an impaired Hsp70 system and the UPS system on a stringent Hsp70 substrate, we expressed NLS-GFP-Luciferase-DM in the diverse yeast mutant strains. Luciferase-DM harbours two mutations (R188Q/R261Q) and represents a hyper-thermolabile variant, sensitizing the substrate towards Hsp70 capacity (Gupta et al., 2011). The GFP-tagged substrate was targeted to the nucleus by fusing the SV40 nuclear localization sequence (NLS) since *ΔΔbtn2Δ* cells are lacking the nuclear sequestrase Btn2 and are particularly vulnerable in this compartment (Ho et al., 2019).

**FIGURE 2:**
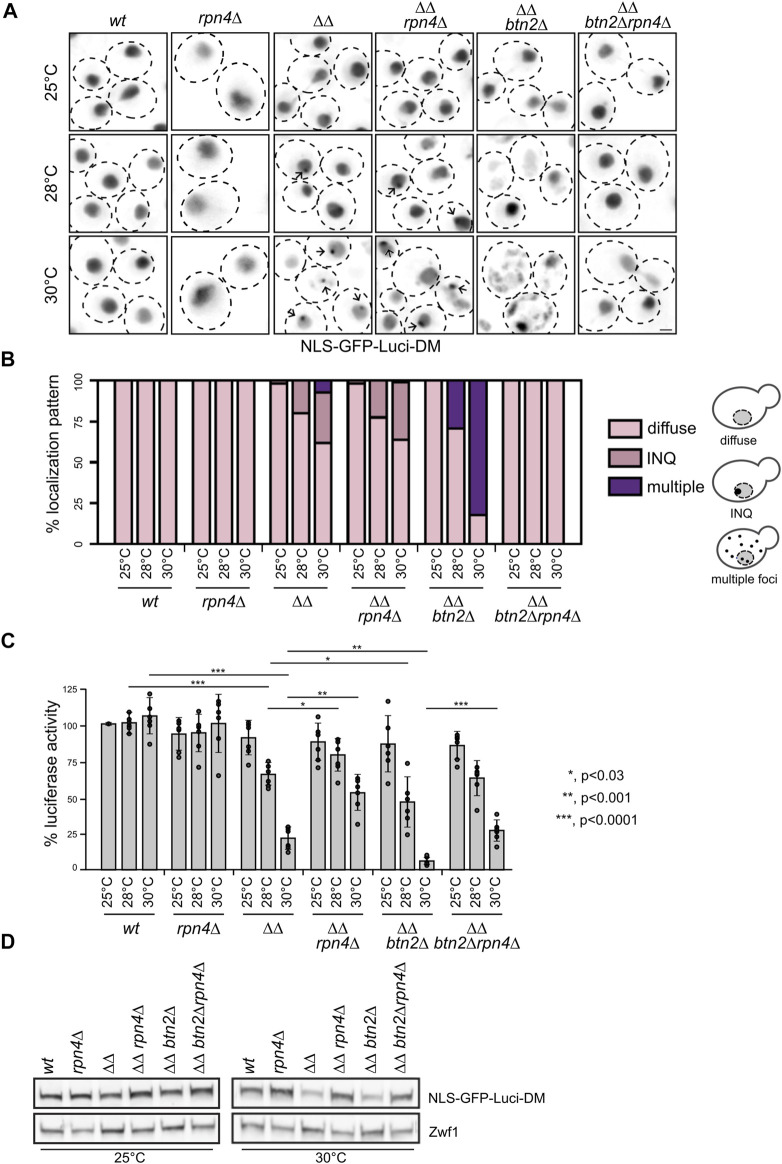
Reducing UPS activity in Hsp70 capacity mutants prevents exhaustive degradation and aggregation of the thermolabile NLS-GFP-Luci-DM proteostasis reporter. **(A)** *S. cerevisiae* wild type (wt) and chaperone mutant cells (*ΔΔ: hsp104Δ fes1Δ*) expressing NLS-GFP-Luci-DM were grown at indicated temperatures. Cellular localization of NLS-GFP-Luci-DM was analyzed by fluorescence microscopy. Maximum intensity Z-projection images of selected cells are shown. Arrows illustrate INQ formation. Scale bar: 2 µm. **(B)** Cellular localizations of NLS-GFP-Luci-DM in respective yeast cells were quantified (n > 50) and categorized into diffuse staining, sequestration at INQ and multiple inclusions (>3 foci/cell). The cartoon representation illustrates the different phenotypes scored. **(C)** Yeast cells were grown as described in **(A)**. Luciferase activities were determined and the activity of WT cells grown at 25°C set to 100%. Standard deviations were calculated based on at least three replicates. An unpaired *t*-test (two-tailed) was used to assess the statistical significance of differences in Luciferase activities between yeast cells (wt and mutants) at 28°C or 30°C. Significance tests for temperature effects are provided in Supplementary Table S3. **(D)** Levels of NLS-GFP-Luci-DM were determined in indicated yeast cells grown at 25°C or 30°C using GFP-specific antibodies. Levels of Zwf1 are provided as loading control.

We first determined the cellular localization of NLS-GFP-Luciferase-DM in the diverse yeast cells between 25°C and 30°C (Figures 2A,B). While the substrate showed a diffuse fluorescence in the nucleus of wt and *rpn4Δ* cells, it formed nuclear foci (INQ) in approx. 31% of *ΔΔ* and 36% of *ΔΔrpn4Δ* cells at 30°C, which is caused by Btn2-mediated sequestration. Accordingly, distinct nuclear foci were no longer observed upon deletion of *btn2*, which instead caused uncontrolled aggregation of the reporter throughout the cell in 30% and 82% of *ΔΔbtn2Δ* cells at 28°C and 30°C (Figures 2A,B). Remarkably, the cellular localization of NLS-GFP-Luciferase-DM was reverted to a diffuse, wt-like appearance in *ΔΔbtn2Δrpn4Δ* and multiple aggregates indicative or PQC collapse were no longer observed. This indicates a major restoration of proteostasis in *ΔΔbtn2Δrpn4Δ* cells.

We next determined the activity of NLS-GFP-Luciferase-DM in the diverse yeast strains grown at 25°C–30°C. While the Luciferase activities remained unaffected by increased growth temperatures in wt and *rpn4Δ* cells, we observed a 4-fold and 15-fold reduction in *ΔΔ* and *ΔΔbtn2Δ* cells at 30°C compared to 25°C (Figure 2C). This documents an increasingly severe loss of Hsp70 function, resulting in misfolding of the Hsp70 substrate. NLS-GFP-Luciferase-DM activities increased by 2.4-fold and 4.1-fold (30°C) when the *rpn4* deletion was linked to *ΔΔ* and *ΔΔbtn2* cells, respectively (Figure 2C). This finding suggests that an imbalance between Hsp70 refolding and proteolytic UPS activities exists in *ΔΔ* and *ΔΔbtn2Δ* leading to enhanced degradation of the reporter. Indeed, NLS-GFP-Luciferase-DM levels were specifically lowered in *ΔΔ* and *ΔΔbtn2Δ* cells at 30°C but re-increased upon additional deletion of *rpn4* (Figure 2D). These finding indicate that lowering UPS activity in *ΔΔbtn2Δ* cells increases proteostasis capacity preventing uncontrolled reporter aggregation and additionally protects the Luciferase from degradation, ultimately increasing reporter activity.

### Induction and attenuation of stress responses in *ΔΔbtn2Δ* and *ΔΔbtn2Δrpn4Δ* cells

**FIGURE 3:**
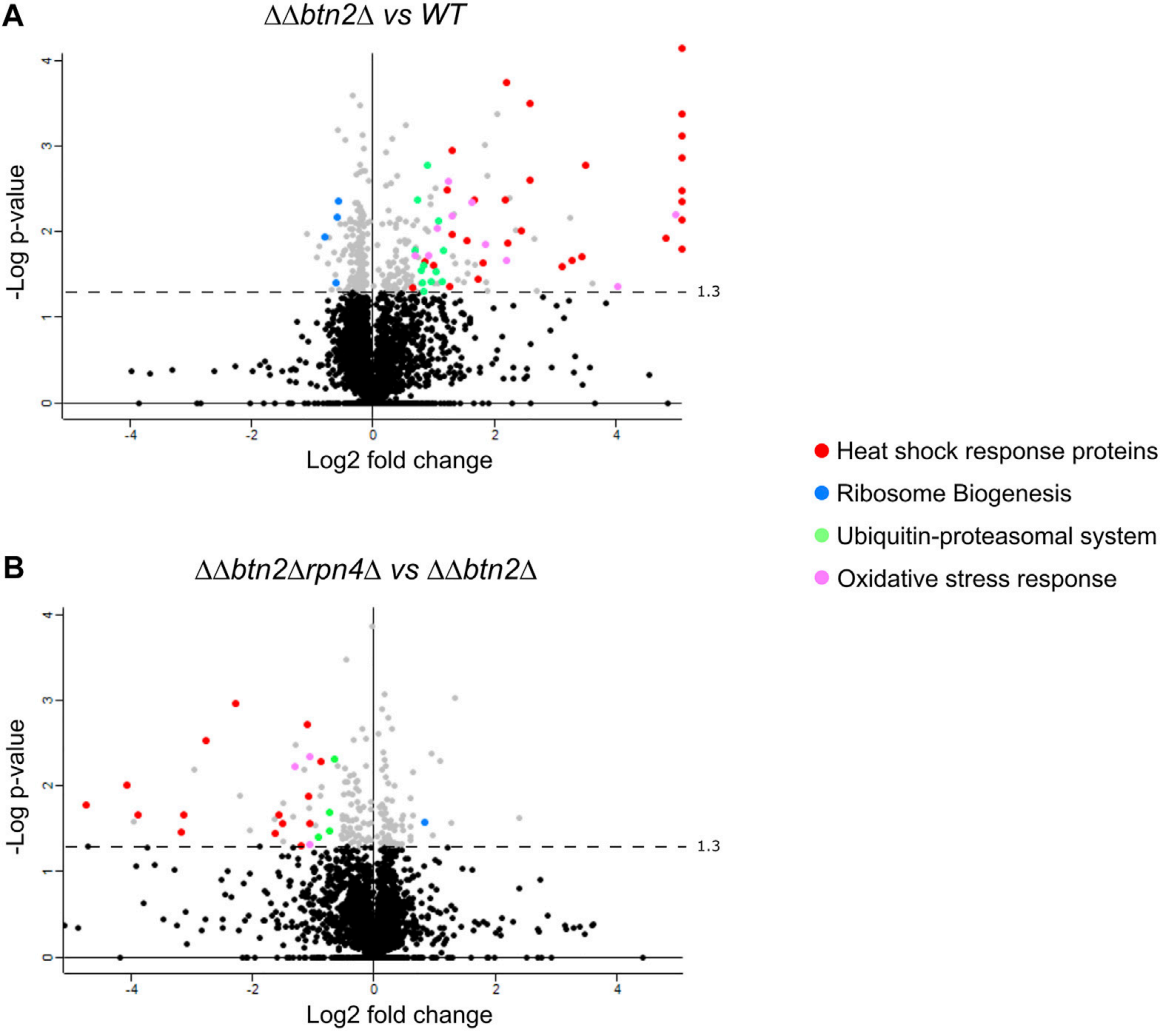
Global effects of proteostasis mutants on protein expression. (A/B) Protein levels were compared between *ΔΔbtn2Δ* (*ΔΔ: hsp104Δ fes1Δ*) and *ΔΔbtn2Δrpn4Δ* **(A)** and *ΔΔbtn2Δ* cells **(B)** at 30°C. For each protein, the *x* axis show the average log_2_ fold change and the *y* axis shows the *p*-value for that difference calculated by *t*-test (two-tailed; n = 2). Proteins belonging to the indicated functional pathways and being significantly up- or downregulated (threshold: 1.5-fold) are highlighted. Proteins lining up on the right border **(A)** are upregulated at least 5-fold The dashed lines indicate the *p*-value threshold of 0.05.

To dissect the cellular consequences but also strategies to deal with a strongly depleted Hsp70 capacity we determined the proteome-wide changes triggered in *ΔΔ* and *ΔΔbtn2Δ* cells by SILAC experiments (Figure 3A, Supplementary Figure S2). For data analysis we set a threshold of factor two for proteins being either enriched or disenriched in the mutant cells at 30°C as compared to wt. We observed a strong upregulation of approx. 25 heat shock proteins in *ΔΔbtn2Δ* and *ΔΔ* cells including all Hsp70 members Ssa1-4 (Figure 3A, Supplementary Figures S2A, S5). The heat shock genes are controlled by the transcription factors Hsf1 and Msn2/4 (Figure S6A). Hsf1 is negatively regulated in yeast cells by Hsp70 (Zheng et al., 2016), rationalizing the upregulation of heat shock genes. Indeed 13 of the 26 heat shock genes upregulated in *ΔΔbtn2Δ* cells are predominantly controlled by Hsf1 (Solis et al., 2018). *ΔΔ* cells also showed an increase in levels of UPS proteins, which was further enhanced in *ΔΔbtn2Δ* cells. Notably, we also observed reduced levels of proteins involved in ribosome production and biogenesis and this reduction was particularly pronounced for *ΔΔbtn2* cells (Figure 3A).

**FIGURE 4:**
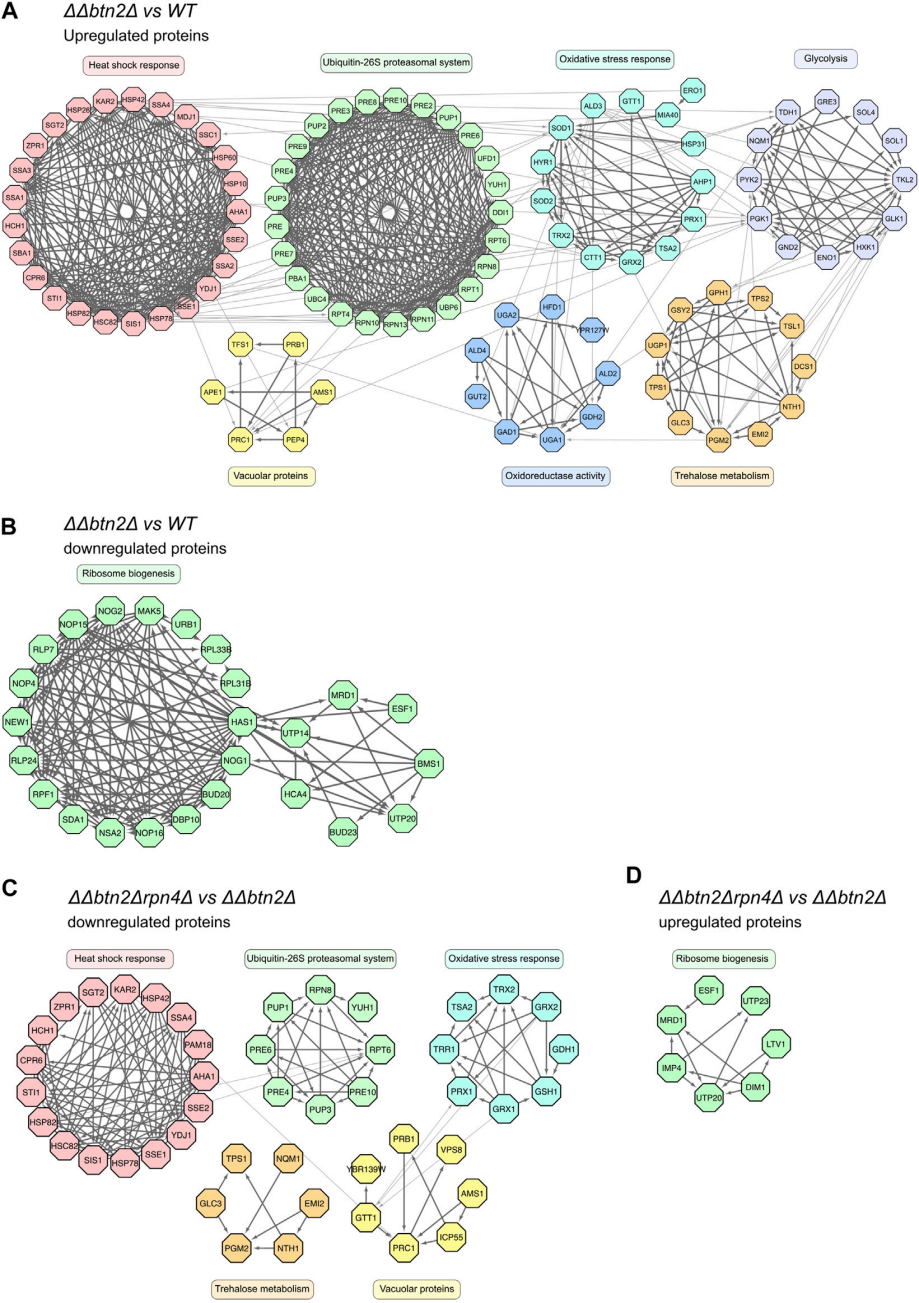
Yeast mutants with strongly reduced Hsp70 capacity activate multiple stress response pathways while downregulating ribosome biogenesis. **(A–D)**: Upregulated (>2-fold) or downregulated (<2-fold) proteins of indicated strain comparisons were clustered according to a common cellular function. Cluster functions and members and their interconnections are indicated.

To better visualize the data and identify which cellular pathways are changed in the *ΔΔbtn2Δ* and *ΔΔ* mutant cells we analyzed the 1.5-fold up- or downregulated proteins for being part of functional clusters defined through their interconnections in the STRING-database. We only considered clusters with at least five proteins in order to focus on the most relevant pathways affected in the mutants (Figure 4). Consistent with the volcano plot analysis, the largest functional clusters induced in *ΔΔbtn2Δ* cells represented proteins linked to the heat shock response and the UPS system with each cluster including 25 members (Figure 4A). The third large cluster of upregulated proteins with 13 members is linked to the oxidative stress response. We also noticed a cluster linked to Trehalose metabolism (11 members) and vacuolar proteins (6 members). Trehalose functions as chemical chaperone and can protect misfolded proteins from aggregation (Singer and Lindquist, 1998). Increased expression of biosynthetic enzymes for trehalose is also observed upon heat shock, underlining the stress-protective function of the carbohydrate (Muhlhofer et al., 2019). We therefore speculate that the upregulation of the trehalose cluster represents an additional effort of the proteostasis network to compensate for the very low Hsp70 capacity. We furthermore observed a cluster linked to glycolysis and oxidoreductase activity. The latter is in parts linked to glycerol metabolism, which has been linked to stress protection in yeast cells (Klein et al., 2017). Cluster analysis of upregulated proteins in *ΔΔ* cells revealed a similar outcome with a large heat shock response cluster (24 members) (Supplementary Figure S3A). Notably, the number of members of the UPS cluster (Tyanova and Cox, 2018) and the oxidative stress response cluster (Peters et al., 2015) were lower as compared to *ΔΔbtn2Δ* cells (Supplementary Figures S3A,B). Similarly, we did not notice the appearance of a cluster for Trehalose metabolism and enzymes with oxidoreductase activity in *ΔΔ* cells. Differences in cluster sizes and appearances between *ΔΔ* and *ΔΔbtn2* cells can be explained by enhanced Hsp70 capacity depletion in *ΔΔbtn2Δ*, likely resulting in increased protein damage and enhanced induction of compensatory stress responses.

In case of proteins that were downregulated in *ΔΔbtn2Δ* cells we identified a single large cluster (25 members) linked to ribosome biogenesis (Figure 4B, Supplementary Figure S5). This cluster was much smaller (6 members) in *ΔΔ* cells (Supplementary Figures S3B,C), indicating a much stronger cellular response to reduce ribosome production in *ΔΔbtn2Δ* cells.

We next performed SILAC analysis of *ΔΔbtn2Δrpn4Δ* cells to determine the changes in protein levels associated with the observed growth rescue. We observed a reduction in levels of proteins linked to the UPS system as compared to *ΔΔbtn2Δ*, which is explained by loss of the Rpn4 transcription factor (Figure 4C, Supplementary Figure S4). As consequence no UPS cluster is found when comparing *ΔΔbtn2Δrpn4Δ* and wt cells, documenting the impact of the *rpn4* deletion on expression of UPS components (Supplementary Figure S3B, S4). Notably, when comparing *ΔΔbtn2Δrpn4Δ with ΔΔbtn2Δ* we also observed a decrease in heat shock protein levels (Figure 4C, Supplementary Figure S4). This is somewhat unexpected since the limitations of the Hsp70 chaperone system remain unchanged due to absence of the Fes1/Hsp104 partner proteins and the Btn2 sequestrase (Figure 4C, Supplementary Figure S4). Heat shock protein levels were still increased as compared to wt cells (Supplementary Figure S3D, S4), showing that the heat shock response remains partially activated in *ΔΔbtn2Δrpn4Δ* cells. Further clusters of downregulated proteins included the oxidative stress response, vacuolar proteins and trehalose metabolism (Figure 4C). Together these findings indicate a global attenuation of stress responses in *ΔΔbtn2Δrpn4Δ*. Along the same line, the ribosome biogenesis cluster was upregulated in *ΔΔbtn2Δrpn4Δ* when compared to *ΔΔbtn2Δ* cells (Figure 4D, Supplementary Figure S5). This further stresses that the cellular measures to deal with Hsp70 depletion in *ΔΔbtn2Δ* cells are largely mitigated upon reducing UPS activity by *rpn4* deletion. We infer that the growth rescue upon reducing UPS activity is linked to global restoration of protein homeostasis and attenuation of diverse stress responses.

### Rpn4 deletion alleviates activation of the heat shock response in *ΔΔbtn2Δ* cells

**FIGURE 5:**
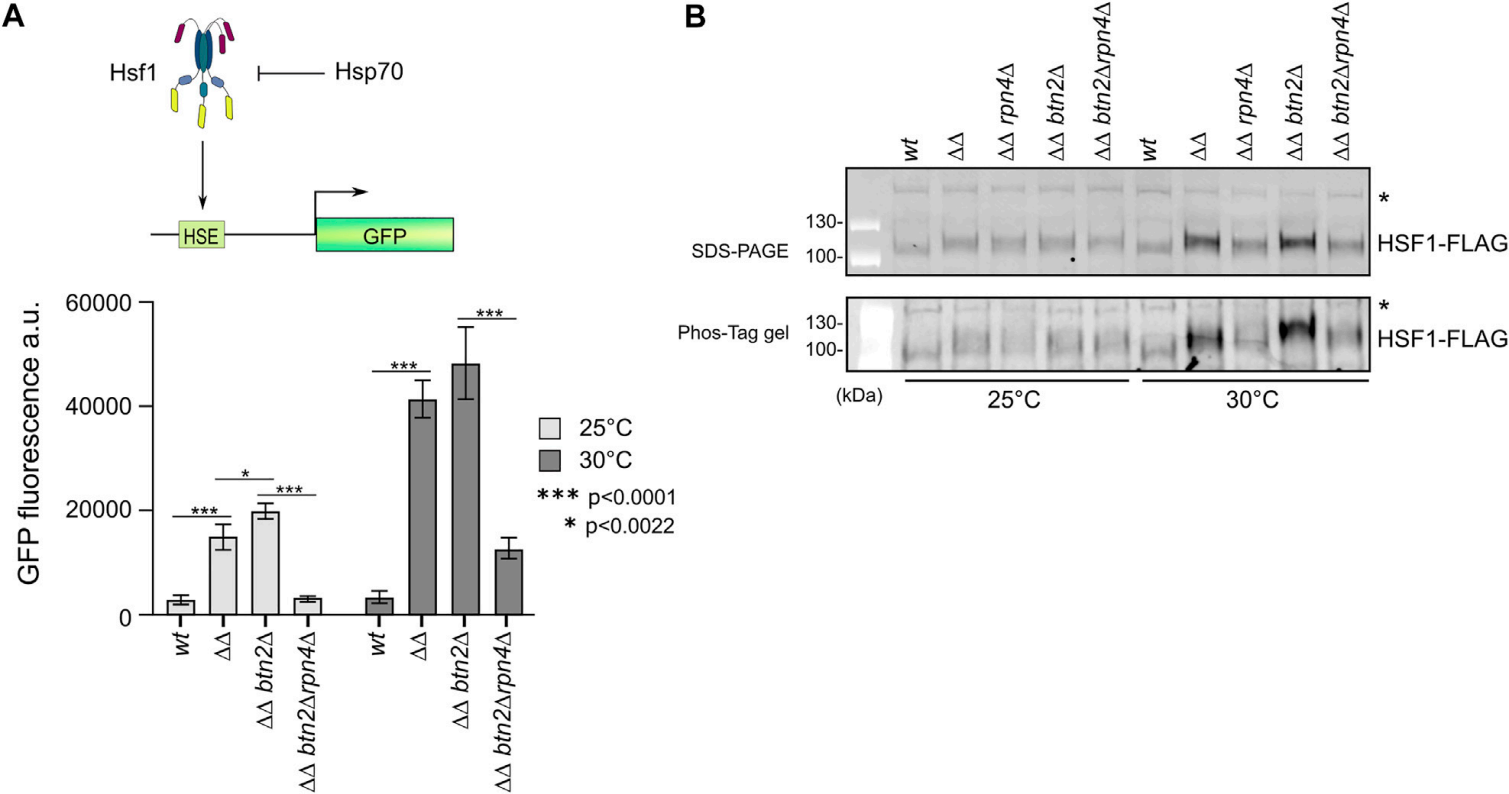
Reducing UPS activity in yeast Hsp70 capacity mutants attenuates Hsf1 activity. **(A)** The reporter gene GFP was placed under the control of the Hsf1-dependent btn2 promoter. GFP additionally harboured a Btn2-derived degron to increase sensitivity. Indicated yeast cells (*ΔΔ: hsp104Δ fes1Δ*) harbouring the GFP reporter were grown at 25°C or 30°C to mid-log growth phase. GFP fluorescence was quantified by flow cytometry and standard deviations were calculated based on three independent experiments. An unpaired *t*-test (two-tailed) was used to assess the statistical significance of differences in GFP fluorescence intensities at 25°C or 30°C. **(B)** Indicated yeast cells (*ΔΔ: hsp104Δ fes1Δ*) expressing functional Hsf1-FLAG were grown at 25°C or 30°C to mid-log growth phase. Total protein extracts were generated and separated by SDS-PAGE and a Phos-Tag gel, increasing the differences in running positions between phosphorylated and non-phosphorylated proteins. Hsf1 levels and phosphorylation states were determined by western blot analysis using FLAG-specific antibodies. “*” indicates non-specific, cross-reacting band that serves as loading control.

**FIGURE 6:**
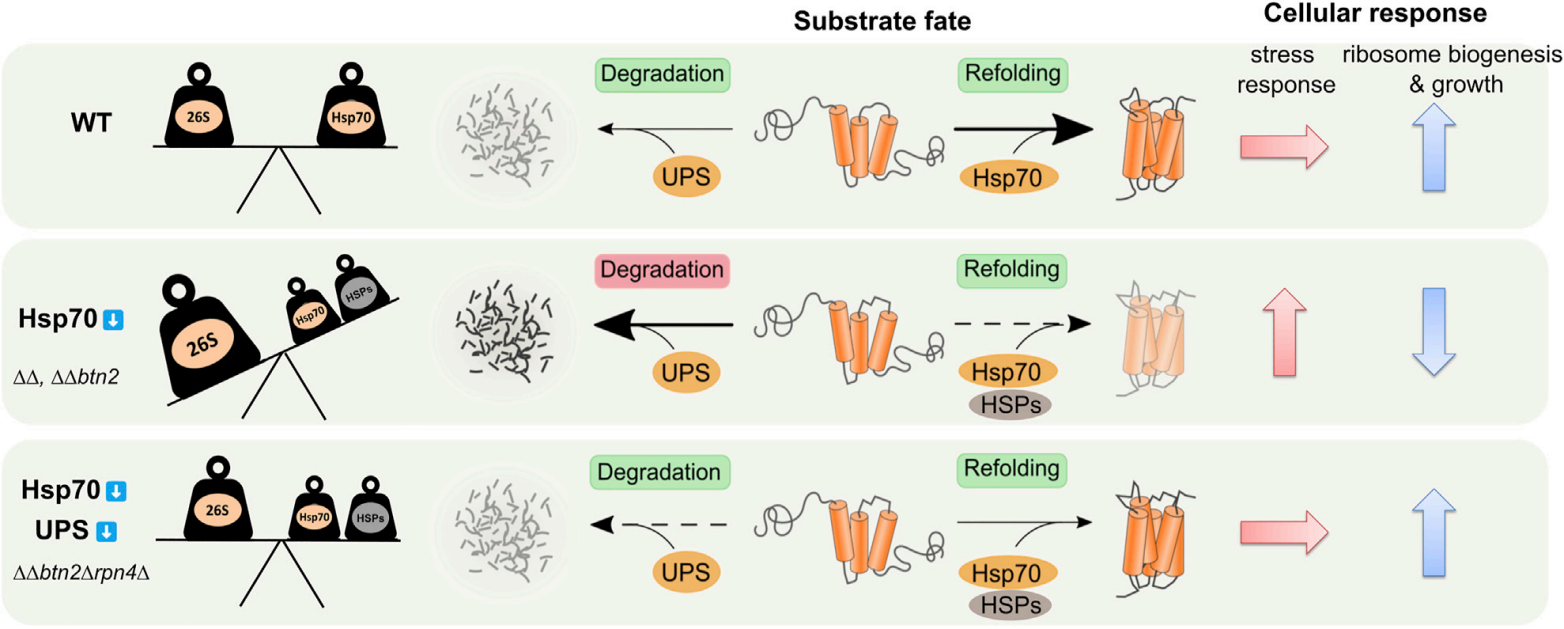
Balancing of Hsp70 and UPS activities is crucial for protein homeostasis. Hsp70 capacity mutants *ΔΔ* (*ΔΔ: hsp104Δ fes1Δ*) and *ΔΔbtn2Δ* suffer from overzealous UPS (26S) activity. These cells activate diverse stress response pathways including overproduction of heat shock proteins (HSPs) and UPS components and decrease ribosome biogenesis as compensatory measures. Rebalancing of refolding Hsp70/HSPs and degrading UPS activities in *ΔΔbtn2Δrpn4Δ* cells restores protein homeostasis and ribosome biogenesis and attenuates stress responses.

The members of the heat shock gene cluster which is strongly upregulated in *ΔΔ* and *ΔΔbtn2Δ* cells are controlled by the Hsf1 and Msn2/4 transcription factors (Supplementary Figure S6A). Some of the upregulated chaperones (e.g. Hsc82, Ssa2) are exclusively regulated by Hsf1 (Solis et al., 2018). An increase in Hsf1 activity is explained by low capacity of Hsp70, which is the main negative regulator of Hsf1 in yeast cells (Krakowiak et al., 2018). We confirmed the SILAC experiments by determining the levels of chaperones (e.g. Ssa1-4, Hsp40 co-chaperones Ydj1, Sis1) in the diverse mutant strains by western blot analysis (Supplementary Figure S6B). We observed strongest upregulation (e.g. Hsp42, Hsp26) in *ΔΔbtn2Δ* cells and a substantial reduction in protein levels upon linking the Hsp70 capacity mutants to *rpn4Δ*. To directly document that Hsf1 is responsible for the changing expression profiles we took advantage of a *gfp* reporter gene placed under exclusive control of Hsf1(Figure 5A) (Ho et al., 2019). GFP levels were determined by FACS in the diverse proteostasis mutants at 25°C and 30°C, revealing a GFP expression profile almost identical to SILAC and western blot analysis (Figure 5A). This demonstrates that changes in Hsf1 activity states are responsible for the differing chaperone levels. We finally tested for Hsf1 levels and phosphorylation status, which positively correlates with Hsf1 activity (Krakowiak et al., 2018), by western blot analysis. This was achieved by expressing functional Hsf1-FLAG as sole Hsf1 copy in the diverse mutant strains and probing for Hsf1 using FLAG-specific antibodies (Figure 5B, Supplementary Figure S6C). Notably, we observed in Hsp70 capacity mutants particularly at 30°C an increase in Hsf1 levels and additionally an upshift of the Hsf1 running position, indicating enhanced phosphorylation, which we confirmed by employing Phos-Tag gels. Reducing UPS activity in Hsp70 capacity mutants overrode these effects (Figure 6B). This finding also indicates that the observed differences in Hsf1 levels do not stem from proteasomal degradation. We cannot exclude the possibility that the Hsf1 phoshorylation status indirectly affects western blotting efficiencies.

Together these findings demonstrate that differences in Hsf1 activity states are mainly responsible for the determined alterations of heat shock protein levels in the tested proteostasis mutants and document downregulation of Hsf1 activity upon reducing UPS activity.

## Discussion

In the presented work we set out to dissect the interconnections between the three major proteostasis strategies: refolding, degradation and sequestration of misfolded proteins. Our work builds up on the previously identified link between the Hsp42 and Btn2 sequestrases and the Hsp70 chaperone system, revealing a major role of protein sequestration in protecting cells from Hsp70 overload (Ho et al., 2019). Negative genetic interactions between the sHsp sequestrases of *E. coli* and *C. elegans* and respective Hsp70 systems indicate that this relationship between the sequestration and refolding branches of proteostasis networks is evolutionarily conserved (Mogk et al., 2003; Shrivastava et al., 2022). In the current analysis we focused on the nuclear sequestrase Btn2 as its deletion conferred the strongest growth defects in Hsp70 capacity mutants (Ho et al., 2019).

To study the role of the UPS system in *∆∆btn2∆* cells which suffer from low refolding and sequestration activities, we genetically reduced the levels of functional 26S proteasomes. We observed a partial rescue of the temperature-sensitive growth defect of *∆∆btn2∆* cells instead of proteostasis breakdown. Growth rescue was also observed when employing the same strategy in *∆∆* cells, indicating that UPS activity is in general detrimental to cells with a low Hsp70 capacity. The positive genetic interaction between Btn2 and the UPS system observed in *∆∆btn2∆* cells is thus primarily caused by the severe reduction of Hsp70 capacity. We infer that balancing of proteolytic and Hsp70-dependent refolding activity is crucial for protein homeostasis and the imbalance created in *∆∆* and *∆∆btn2∆* is largely responsible for the proteostasis defects (Figure 6). In this context the increased Hsf1-dependent expression of other refolding chaperone machineries (e.g. Hsp90) might also contribute to maintain the balance and to rescue the Hsp70 capacity mutants (Figure 6). These findings are in line with an initial report by Craig and others, that identified the deubiquitinating enzyme Ubp3 as high-copy suppressor of the ts-phenotype of *ssa1∆ssa2∆* cells (Baxter and Craig, 1998). Similarly, Nystrom and others observed synthetic lethality when combining *ssa1∆ ssa2∆* with *ubr2∆*. The E3 ligase Ubr2 is mainly responsible for Rpn4 degradation, suggesting that boosting proteasomal activity in Hsp70 mutants creates toxicity (Oling et al., 2014). We directly demonstrate an overzealous protein quality control system in *∆∆* and *∆∆btn2∆* cells by determining reduced levels of the stringent Hsp70 substrate NLS-GFP-Luci-DM (Figure 2). Reducing UPS activity in the Hsp70 capacity mutants re-increased Luciferase levels and also partly its enzymatic activity, indicating that a restored balance of both activities prevents exhaustive protein loss through proteolysis.

SILAC analysis of *∆∆* and *∆∆btn2∆* cells uncovered the various cellular strategies to compensate for the loss of Hsp70 capacity. We observed an activation of diverse stress responses, including the Hsf1-dependent heat shock response, the Msn2/4 and Yap1 controlled oxidative stress response (Supplementary Figure S6A) and the Rpn4 controlled proteasome stress response (PSR) (Work and Brandman, 2021). Furthermore, we noticed increased levels of proteins linked to glycolysis and trehalose metabolism, responses that are also triggered in yeast cells upon heat shock (Muhlhofer et al., 2019). Stress responses were strongest upregulated in *∆∆btn2∆* cells, reflecting an aggravated loss of Hsp70 capacity in these cells and enhanced cellular attempts to compensate for the defect. Our findings are similar to the hyper-stress program, which is mounted in heat shocked *fes1∆* cells (Masser et al., 2019), confirming that the persistent depletion of Hsp70 causes hyperinduction of Hsf1 activity. Strongest defects of *∆∆btn2∆* cells are also apparent from the specific downregulation of ribosome biogenesis factors (Supplementary Figure S3B). Expression of these factors is controlled by Sf1p (Supplementary Figure S6A), which links ribosome production and biogenesis to growth rates (Albert et al., 2019). The reduced growth rate of *∆∆btn2∆* cells might therefore also contribute to reduced production of the biogenesis factors. We also consider that this depletion represents a cellular attempt to reduce the influx of nascent polypeptides into the proteostasis network relieving chaperone systems involved in the folding of nascent polypeptides including the Hsp70 members Ssa1/2 (Kim et al., 1998). The downregulation of translation factors is an evolutionarily conserved strategy and observed in yeast, *C. elegans* and human cell cultures upon stress application (Kirstein-Miles et al., 2013; Liu et al., 2013; Boos et al., 2019; Muhlhofer et al., 2019) and reduction of translational capacity can have beneficial effects on the proteostasis network of stressed cells (Kirstein-Miles et al., 2013).

While the upregulation of cellular stress responses might enable survival of *∆∆* and *∆∆btn2∆* cells at moderate growth temperatures they can also exert detrimental effects as shown here for induction of the UPS system. Enhanced UPS and Rpn4 activities in *∆∆* and *∆∆btn2∆* cells can be explained in two ways. First, accumulation of misfolded proteins can act as competing substrates leading to stabilization of the UPS target Rpn4 (Xie and Varshavsky, 2001). Second, *rpn4* expression is directly controlled by Hsf1 (Hahn et al., 2006). The enhanced Hsf1 activity that we document in *∆∆* and *∆∆btn2∆* cells (Figure 5) thus directly results in UPS upregulation. Accordingly, heat shock also leads to induction of UPS components (Muhlhofer et al., 2019). Notably, UPS induction is more pronounced at severe heat stress (42°C) and is temporally shifted after enhanced chaperone expression, suggesting a cellular strategy to prioritize protein folding over degradation (Muhlhofer et al., 2019). While the dual induction of refolding and proteolytic activities might be beneficial during acute stress conditions, it is detrimental in *∆∆* to *∆∆btn2∆* cells, which suffer from chronic stress due to permanent loss of Hsp70 capacity.

Reducing UPS in *∆∆* and *∆∆btn2∆* cells does not only protect from uncontrolled proteolysis but, remarkably, also largely restores protein homeostasis (Figure 2) and globally attenuates stress responses activated in the Hsp70 mutant cells (Figure 4). This suggests massive collateral damage in *∆∆* and *∆∆btn2∆* cells caused by overhasty protein degradation and ultimately triggering a diverse set of stress responses. How *∆∆btn2∆rpn4∆* cells downregulate Hsf1 activity remains unclear as these cells still suffer from low Hsp70 capacity. We noticed reduced phosphorylation of Hsf1 upon *rpn4* deletion, eventually pointing to a protein phosphatase, which acts as negative Hsf1 regulator and becomes stabilized upon lowering UPS activity. Our SILAC analysis, however, did not reveal increased levels of such factor, leaving the mechanistic basis for Hsf1 activity attenuation unsolved.

Taken together our findings indicate opposing roles of Hsp70 and UPS functions in the proteostasis network of yeast cells. These activities cannot functionally replace each other but they need to be precisely adjusted and balanced. This interplay between Hsp70 and UPS activity is different from other proteostasis interconnections. For example, an increase in UPS activity rescues yeast cells suffering from accumulation of ER proteins in the cytosol due to impaired translocation (Schmidt et al., 2019). This opposing effect can be explained by the inability of ER proteins to fold in the cytosol and their UPS-mediated degradation will thus prevent overload of e.g. Hsp70 chaperones. The interconnections in the proteostasis network will therefore depend on the specific cellular context and stress conditions applied and the resulting burden on the PQC system.

## Data Availability

The datasets presented in this study can be found in online repositories. The names of the repository/repositories and accession number(s) can be found below: https://www.ebi.ac.uk/pride/archive/, PXD037835.

## References

[B1] Albert B. Tomassetti S. Gloor Y. Dilg D. Mattarocci S. Kubik S. (2019). Sfp1 regulates transcriptional networks driving cell growth and division through multiple promoter-binding modes. Genes. Dev. 33 (5-6), 288–293. 10.1101/gad.322040.118 30804227 PMC6411004

[B2] Barenz F. Inoue D. Yokoyama H. Tegha-Dunghu J. Freiss S. Draeger S. (2013). The centriolar satellite protein SSX2IP promotes centrosome maturation. J. Cell. Biol. 202 (1), 81–95. 10.1083/jcb.201302122 23816619 PMC3704989

[B3] Baxter B. K. Craig E. A. (1998). Isolation of UBP3, encoding a de-ubiquitinating enzyme, as a multicopy suppressor of a heat-shock mutant strain of *S. cerevisiae* . Curr. Genet. 33 (6), 412–419. 9644204 10.1007/s002940050354

[B4] Boos F. Kramer L. Groh C. Jung F. Haberkant P. Stein F. (2019). Mitochondrial protein-induced stress triggers a global adaptive transcriptional programme. Nat. Cell. Biol. 21 (4), 442–451. 10.1038/s41556-019-0294-5 30886345

[B5] den Brave F. Cairo L. V. Jagadeesan C. Ruger-Herreros C. Mogk A. Bukau B. (2020). Chaperone-mediated protein disaggregation triggers proteolytic clearance of intra-nuclear protein inclusions. Cell. Rep. 31 (9), 107680. 10.1016/j.celrep.2020.107680 32492414 PMC7273177

[B6] Escusa-Toret S. Vonk W. I. Frydman J. (2013). Spatial sequestration of misfolded proteins by a dynamic chaperone pathway enhances cellular fitness during stress. Nat. Cell. Biol. 15 (10), 1231–1243. ncb2838 [pii]. 10.1038/ncb2838 24036477 PMC4121856

[B7] Finley D. Prado M. A. (2020). The proteasome and its network: Engineering for adaptability. Cold Spring Harb. Perspect. Biol. 12 (1), a033985. 10.1101/cshperspect.a033985 30833452 PMC6829053

[B8] Glover J. R. Lindquist S. (1998). Hsp104, Hsp70, and Hsp40: A novel chaperone system that rescues previously aggregated proteins. Cell. 94, 73–82. 10.1016/s0092-8674(00)81223-4 9674429

[B9] Gupta R. Kasturi P. Bracher A. Loew C. Zheng M. Villella A. (2011). Firefly luciferase mutants as sensors of proteome stress. Nat. Methods 8 (10), 879–884. nmeth.1697 [pii]. 10.1038/nmeth.1697 21892152

[B10] Hahn J. S. Neef D. W. Thiele D. J. (2006). A stress regulatory network for co-ordinated activation of proteasome expression mediated by yeast heat shock transcription factor. Mol. Microbiol. 60 (1), 240–251. 10.1111/j.1365-2958.2006.05097.x 16556235

[B11] Ho C. T. Grousl T. Shatz O. Jawed A. Ruger-Herreros C. Semmelink M. (2019). Cellular sequestrases maintain basal Hsp70 capacity ensuring balanced proteostasis. Nat. Commun. 10 (1), 4851. 10.1038/s41467-019-12868-1 31649258 PMC6813348

[B12] Houck S. A. Ren H. Y. Madden V. J. Bonner J. N. Conlin M. P. Janovick J. A. (2014). Quality control autophagy degrades soluble ERAD-resistant conformers of the misfolded membrane protein GnRHR. Mol. Cell. 54 (1), 166–179. 10.1016/j.molcel.2014.02.025 24685158 PMC4070183

[B13] Kabani M. Beckerich J. M. Brodsky J. L. (2002). Nucleotide exchange factor for the yeast Hsp70 molecular chaperone Ssa1p. Mol. Cell. Biol. 22 (13), 4677–4689. 10.1128/mcb.22.13.4677-4689.2002 12052876 PMC133915

[B14] Kaganovich D. Kopito R. Frydman J. (2008). Misfolded proteins partition between two distinct quality control compartments. Nature 454 (7208), 1088–1095. 10.1038/nature07195 18756251 PMC2746971

[B15] Kim S. Schilke B. Craig E. A. Horwich A. L. (1998). Folding *in vivo* of a newly translated yeast cytosolic enzyme is mediated by the SSA class of cytosolic yeast Hsp70 proteins. Proc. Natl. Acad. Sci. U. S. A. 95, 12860–12865. 10.1073/pnas.95.22.12860 9789005 PMC23633

[B16] Kirstein-Miles J. Scior A. Deuerling E. Morimoto R. I. (2013). The nascent polypeptide-associated complex is a key regulator of proteostasis. EMBO J. 32 (10), 1451–1468. 10.1038/emboj.2013.87 23604074 PMC3655472

[B17] Klein M. Swinnen S. Thevelein J. M. Nevoigt E. (2017). Glycerol metabolism and transport in yeast and fungi: Established knowledge and ambiguities. Environ. Microbiol. 19 (3), 878–893. 10.1111/1462-2920.13617 27878932

[B18] Krakowiak J. Zheng X. Patel N. Feder Z. A. Anandhakumar J. Valerius K. (2018). Hsf1 and Hsp70 constitute a two-component feedback loop that regulates the yeast heat shock response. Elife 7, e31668. 10.7554/eLife.31668 29393852 PMC5809143

[B19] Kusmierczyk A. R. Kunjappu M. J. Funakoshi M. Hochstrasser M. (2008). A multimeric assembly factor controls the formation of alternative 20S proteasomes. Nat. Struct. Mol. Biol. 15 (3), 237–244. 10.1038/nsmb.1389 18278055

[B20] Lilienbaum A. (2013). Relationship between the proteasomal system and autophagy. Int. J. Biochem. Mol. Biol. 4 (1), 1 23638318 PMC3627065

[B21] Liu B. Han Y. Qian S. B. (2013). Cotranslational response to proteotoxic stress by elongation pausing of ribosomes. Mol. Cell. 49 (3), 453–463. 10.1016/j.molcel.2012.12.001 23290916 PMC3570626

[B22] Mannhaupt G. Schnall R. Karpov V. Vetter I. Feldmann H. (1999). Rpn4p acts as a transcription factor by binding to PACE, a nonamer box found upstream of 26S proteasomal and other genes in yeast. FEBS Lett. 450 (1-2), 27–34. 10.1016/s0014-5793(99)00467-6 10350051

[B23] Marshall R. S. McLoughlin F. Vierstra R. D. (2016). Autophagic turnover of inactive 26S proteasomes in yeast is directed by the ubiquitin receptor Cue5 and the Hsp42 chaperone. Cell. Rep. 16 (6), 1717–1732. 10.1016/j.celrep.2016.07.015 27477278

[B24] Masser A. E. Kang W. Roy J. Kaimal J. M. Quintana-Cordero J. Friedlander M. R. (2019). Cytoplasmic protein misfolding titrates Hsp70 to activate nuclear Hsf1. Elife 8, e47791. 10.7554/eLife.47791 31552827 PMC6779467

[B25] Miller S. B. Ho C. T. Winkler J. Khokhrina M. Neuner A. Mohamed M. Y. (2015). Compartment-specific aggregases direct distinct nuclear and cytoplasmic aggregate deposition. EMBO J. 34 (6), 778–797. 10.15252/embj.201489524 25672362 PMC4369314

[B26] Mizushima N. Noda T. Yoshimori T. Tanaka Y. Ishii T. George M. D. (1998). A protein conjugation system essential for autophagy. Nature 395 (6700), 395–398. 10.1038/26506 9759731

[B27] Mogk A. Deuerling E. Vorderwulbecke S. Vierling E. Bukau B. (2003). Small heat shock proteins, ClpB and the DnaK system form a functional triade in reversing protein aggregation. Mol. Microbiol. 50 (2), 585–595. 10.1046/j.1365-2958.2003.03710.x 14617181

[B28] Muhlhofer M. Berchtold E. Stratil C. G. Csaba G. Kunold E. Bach N. C. (2019). The heat shock response in yeast maintains protein homeostasis by chaperoning and replenishing proteins. Cell. Rep. 29 (13), 4593–4607. 10.1016/j.celrep.2019.11.109 31875563

[B29] Oling D. Eisele F. Kvint K. Nystrom T. (2014). Opposing roles of Ubp3-dependent deubiquitination regulate replicative life span and heat resistance. EMBO J. 33 (7), 747–761. 10.1002/embj.201386822 24596250 PMC4000091

[B30] Peters L. Z. Karmon O. David-Kadoch G. Hazan R. Yu T. Glickman M. H. (2015). The protein quality control machinery regulates its misassembled proteasome subunits. PLoS Genet. 11 (4), e1005178. 10.1371/journal.pgen.1005178 25919710 PMC4412499

[B31] Rosenzweig R. Nillegoda N. B. Mayer M. P. Bukau B. (2019). The Hsp70 chaperone network. Nat. Rev. Mol. Cell. Biol. 20 (11), 665–680. 10.1038/s41580-019-0133-3 31253954

[B32] Schmidt R. M. Schessner J. P. Borner G. H. Schuck S. (2019). The proteasome biogenesis regulator Rpn4 cooperates with the unfolded protein response to promote ER stress resistance. Elife 8, e43244. 10.7554/eLife.43244 30865586 PMC6415940

[B33] Shrivastava A. Sandhof C. A. Reinle K. Jawed A. Ruger-Herreros C. Schwarz D. (2022). The cytoprotective sequestration activity of small heat shock proteins is evolutionarily conserved. J. Cell. Biol. 221 (10), e202202149. 10.1083/jcb.202202149 36069810 PMC9458469

[B34] Singer M. A. Lindquist S. (1998). Multiple effects of trehalose on protein folding *in vitro* and *in vivo* . Mol. Cell. 1, 639–648. 10.1016/s1097-2765(00)80064-7 9660948

[B35] Solis E. J. Pandey J. P. Zheng X. Jin D. X. Gupta P. B. Airoldi E. M. (2018). Defining the essential function of yeast Hsf1 reveals a compact transcriptional program for maintaining eukaryotic proteostasis. Mol. Cell. 69 (3), 534. 10.1016/j.molcel.2018.01.021 29395071 PMC5813686

[B36] Sontag E. M. Samant R. S. Frydman J. (2017). Mechanisms and functions of spatial protein quality control. Annu. Rev. Biochem. 86, 97–122. 10.1146/annurev-biochem-060815-014616 28489421

[B37] Specht S. Miller S. B. Mogk A. Bukau B. (2011). Hsp42 is required for sequestration of protein aggregates into deposition sites in *Saccharomyces cerevisiae* . J. Cell. Biol. 195 (4), 617–629. [pii]. 10.1083/jcb.201106037 22065637 PMC3257523

[B38] Tyanova S. Cox J. (2018). Perseus: A bioinformatics platform for integrative analysis of proteomics data in cancer research. Methods Mol. Biol. 1711, 133–148. 10.1007/978-1-4939-7493-1_7 29344888

[B39] Work J. J. Brandman O. (2021). Adaptability of the ubiquitin-proteasome system to proteolytic and folding stressors. J. Cell. Biol. 220 (3), e201912041. 10.1083/jcb.201912041 33382395 PMC7780722

[B40] Xie Y. Varshavsky A. (2001). RPN4 is a ligand, substrate, and transcriptional regulator of the 26S proteasome: A negative feedback circuit. Proc. Natl. Acad. Sci. U. S. A. 98 (6), 3056–3061. 10.1073/pnas.071022298 11248031 PMC30606

[B41] Zheng X. Krakowiak J. Patel N. Beyzavi A. Ezike J. Khalil A. S. (2016). Dynamic control of Hsf1 during heat shock by a chaperone switch and phosphorylation. Elife 5, e18638. 10.7554/eLife.18638 27831465 PMC5127643

