# Supplementary material for: Balanced activities of Hsp70 and the ubiquitin proteasome system underlie cellular protein homeostasis
Source: Front Mol Biosci. 2023 Jan 4;9:1106477. doi: 10.3389/fmolb.2022.1106477 (PMC9845930; doi:10.3389/fmolb.2022.1106477)
Supplement: Supplementary file 3 [file DataSheet1.PDF]

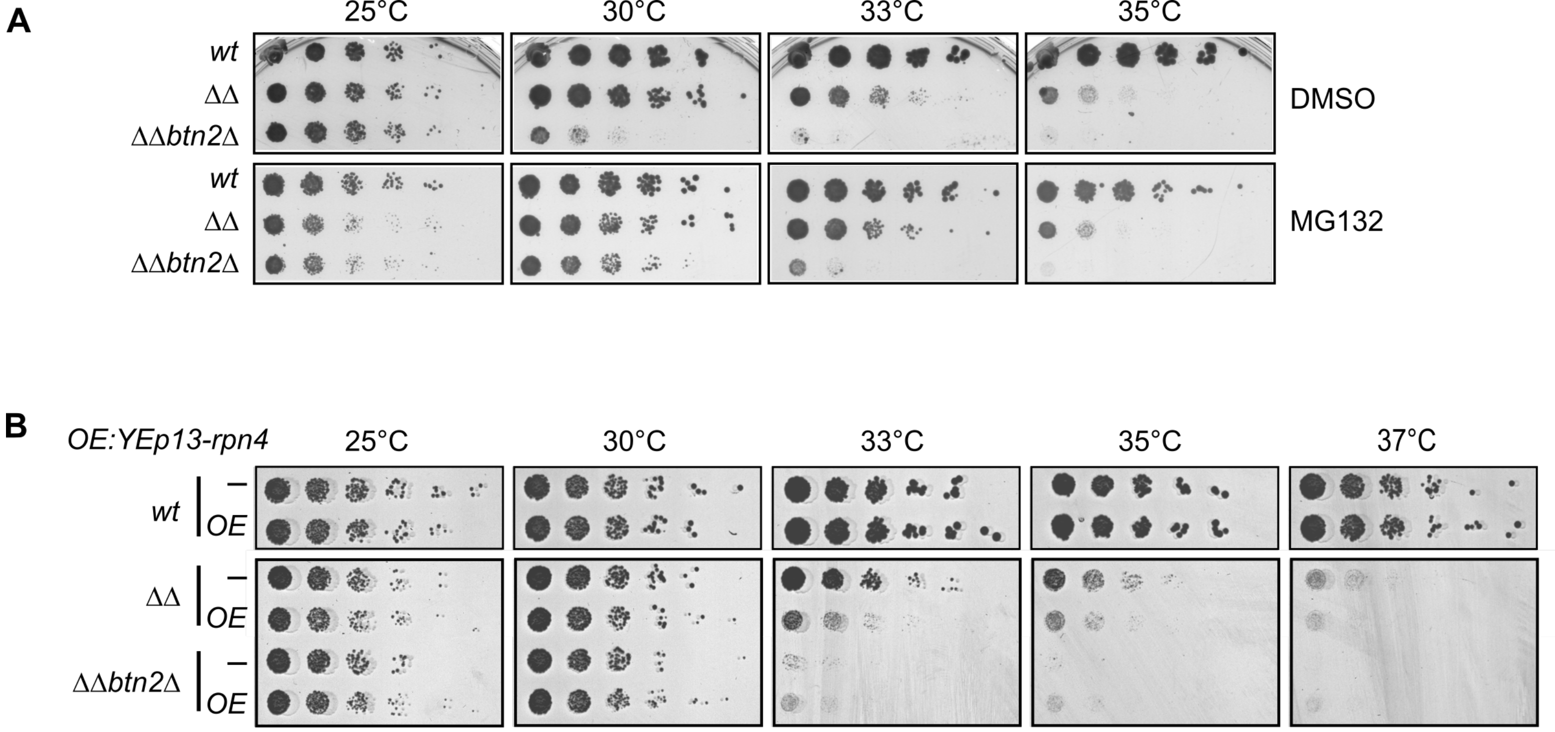
A
25°C
30°C
33°C
35°C
wt
ΔΔ
ΔΔbtn2Δ
DMSO
MG132
B
OE:YEp13-rpn4
37°C
–
OE

Figure S1

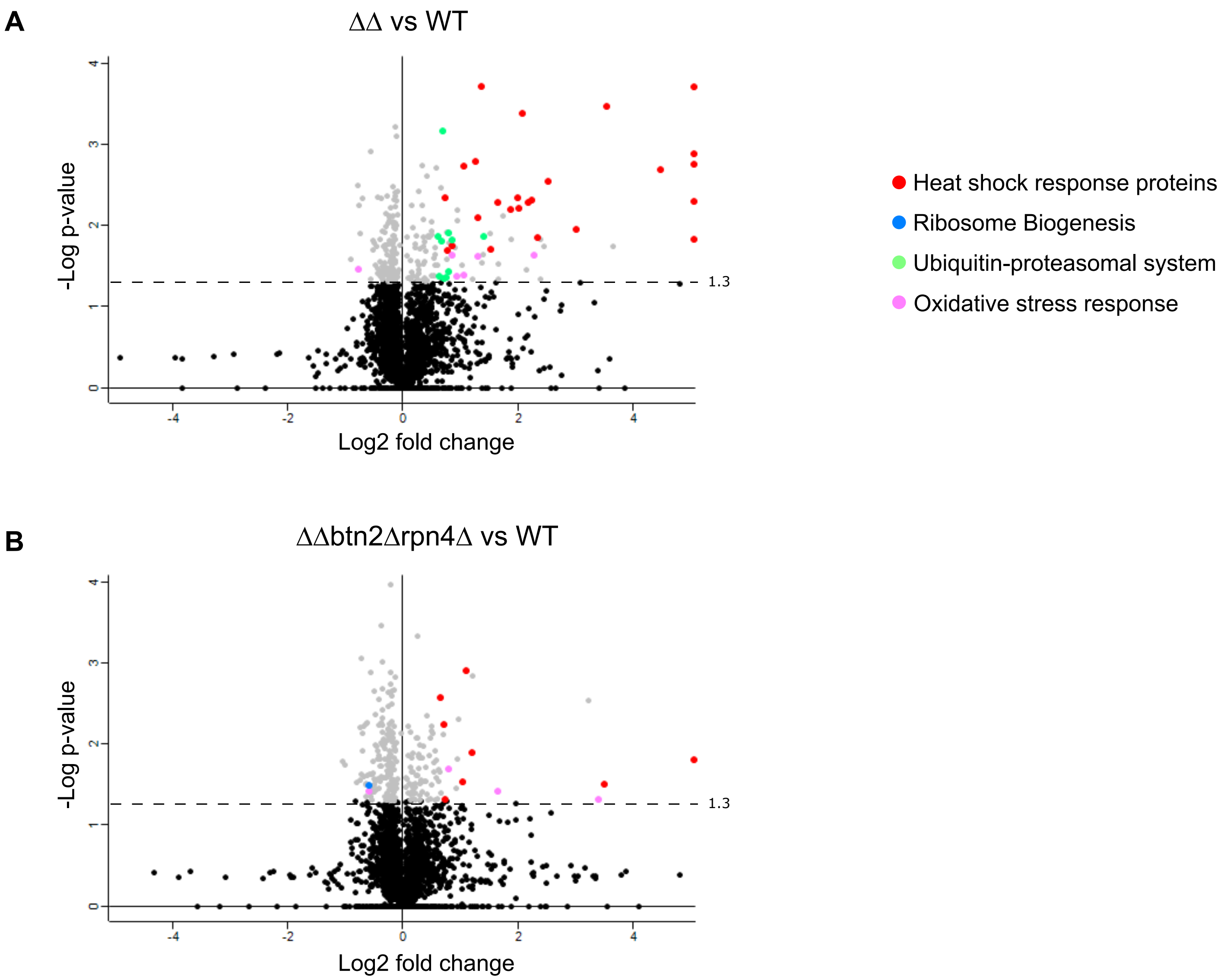
A
ΔΔ vs WT
-Log p-value
Log2 fold change
1.3
B
ΔΔbtn2Δrpn4Δ vs WT
-Log p-value
Log2 fold change
1.3
Heat shock response proteins
Ribosome Biogenesis
Ubiquitin-proteasomal system
Oxidative stress response

Figure S2

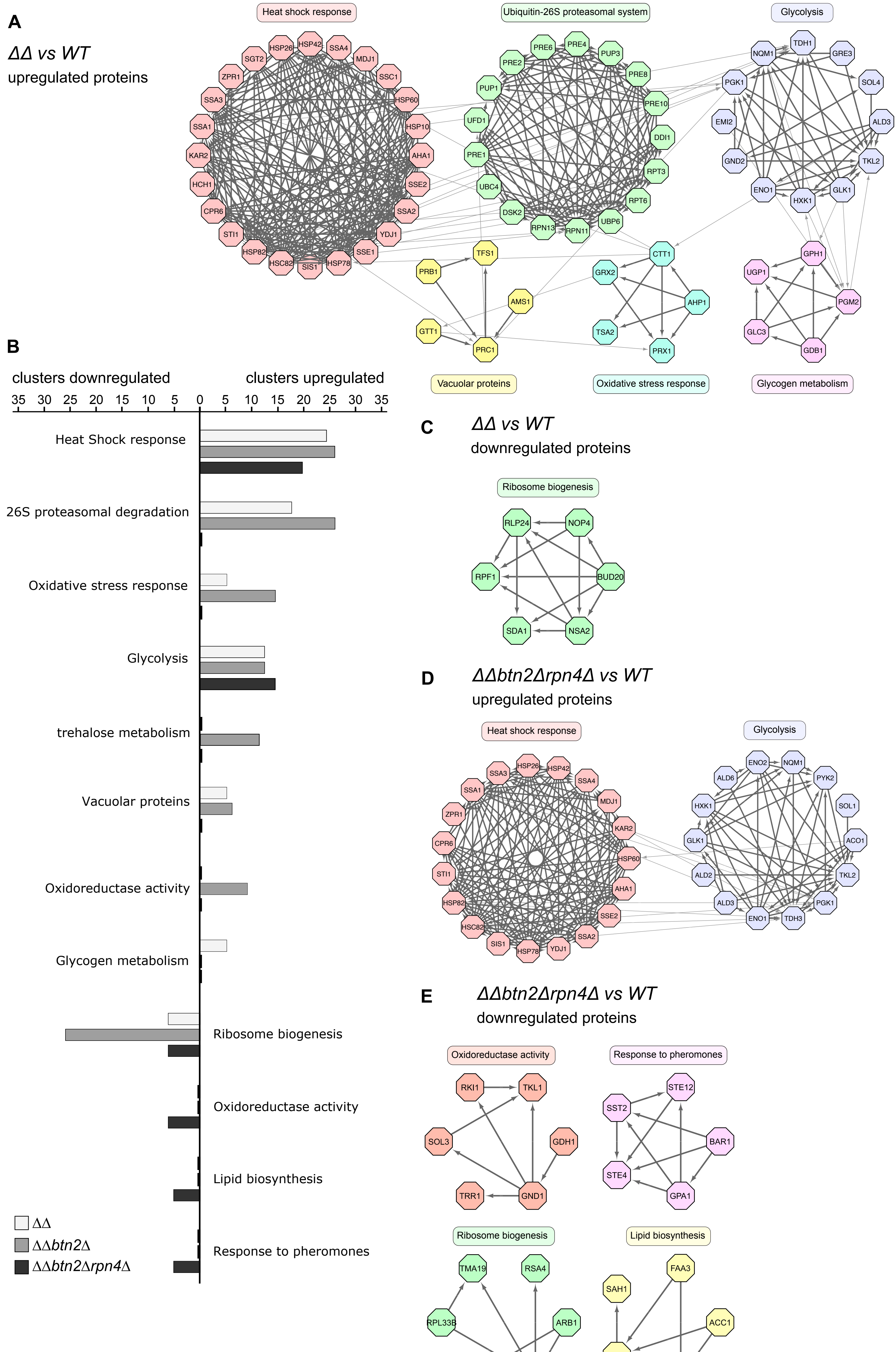
A
ΔΔ vs WT
upregulated proteins
Heat shock response
Ubiquitin-26S proteasomal system
Glycolysis
Vacuolar proteins
Oxidative stress response
Glycogen metabolism
B
clusters downregulated
clusters upregulated
Heat Shock response
26S proteasomal degradation
Oxidative stress response
Glycolysis
trehalose metabolism
Vacuolar proteins
Oxidoreductase activity
Glycogen metabolism
Ribosome biogenesis
Oxidoreductase activity
Lipid biosynthesis
Response to pheromones
ΔΔ
ΔΔbtn2Δ
ΔΔbtn2Δrpn4Δ
C
ΔΔ vs WT
downregulated proteins
Ribosome biogenesis
D
ΔΔbtn2Δrpn4Δ vs WT
upregulated proteins
Heat shock response
Glycolysis
E
ΔΔbtn2Δrpn4Δ vs WT
downregulated proteins
Oxidoreductase activity
Response to pheromones
Ribosome biogenesis
Lipid biosynthesis

Figure S3

	ΔΔ/wt	ΔΔbtn2Δ/wt	ΔΔbtn2Δrpn4Δ/wt
SSA4	5.04±0.05	5.00±0.25	3.74±1.00
HSP42	4.16±0.25	4.77±0.57	3.55±0.86
HSP78	3.39±0.10	3.34±0.13	2.18±0.51
HSP26	3.07±0.22	3.34±0.37	2.45±0.44
SSA3	2.47±0.22	3.29±0.58	2.80±1.14
HSP82	3.13±0.13	3.20±0.22	1.93±0.44
ZPR1	2.91±0.07	2.86±0.11	1.37±0.45
SIS1	2.61±0.03	2.70±0.07	1.56±0.20
SSE2	2.48±0.34	2.62±0.09	1.09±0.30
CPR6	2.47±0.02	2.56±0.13	0.94±0.22
STI1	2.31±0.00	2.30±0.04	0.84±0.09
SSA1	2.11±0.25	2.14±0.34	1.63±0.54
HCH1	1.88±0.26	1.99±0.10	0.40±0.04
AHA1	1.84±0.28	1.87±0.27	1.02±0.01
MDJ1	2.19±0.14	1.85±0.08	1.12±0.23
HSC82	1.81±0.10	1.83±0.01	1.18±0.05
KAR2	1.72±0.19	1.81±0.24	0.86±0.33
HSP10	1.51±0.05	1.58±0.04	0.72±0.55
SGT2	1.44±0.19	1.53±0.04	0.38±0.21
YDJ1	1.61±0.10	1.49±0.07	0.82±0.19
SSE1	1.69±0.01	1.41±0.13	0.59±0.08
HSP60	1.28±0.00	1.24±0.11	0.82±0.19
SSA2	1.35±0.44	1.21±0.30	1.00±0.40
SSC1	1.19±0.07	1.17±0.09	0.67±0.20
SBA1	0.95±0.62	0.74±0.14	0.46±0.73
HSP150	0.39±0.11	0.60±0.02	0.09±0.03

Heat Shock Response

	ΔΔ/wt	ΔΔbtn2Δ/wt	ΔΔbtn2Δrpn4Δ/wt
PUP3	1.07±0.08	1.25±0.20	0.51±0.09
DDI1	1.10±0.08	1.22±0.02	0.74±0.20
PRE3	0.93±0.54	1.22±0.35	0.55±0.21
PUP1	0.97±0.06	1.17±0.40	0.32±0.20
UFD1	0.82±0.12	1.09±0.13	0.39±0.54
PRE4	0.96±0.15	1.07±0.17	0.39±0.19
PRE2	1.25±0.02	1.04±0.18	0.36±0.48
RPN11	0.91±0.16	1.03±0.01	0.61±0.36
PRE10	0.80±0.17	1.03±0.28	0.02±0.00
UBC4	0.75±0.03	0.92±0.00	0.30±0.09
YUH1	1.25±0.02	0.90±0.38	0.13±0.42
PRE7	0.72±0.19	0.90±0.24	0.35±0.28
RPT1	0.76±0.30	0.87±0.30	0.28±0.40
PRE6	0.80±0.24	0.86±0.20	0.10±0.15
PRE8	0.76±0.08	0.84±0.08	0.32±0.15
RPN10	0.60±0.28	0.83±0.27	0.31±0.26
PRE1	0.70±0.03	0.81±0.17	0.23±0.08
RPN13	0.75±0.05	0.79±0.01	0.34±0.05
PBA1	0.66±0.15	0.76±0.15	0.14±0.01
RPT6	0.73±0.19	0.74±0.19	0.02±0.20
RPN8	0.61±0.33	0.69±0.15	0.04±0.22
RPT4	0.57±0.10	0.67±0.04	0.12±0.06
PRE9	0.60±0.05	0.67±0.01	0.17±0.09
UBP6	0.69±0.09	0.63±0.02	0.32±0.25
PUP2	0.57±0.10	0.61±0.01	0.22±0.19
RPT3	0.60±0.01	0.63±0.20	0.22±0.19
DSK2	0.71±0.11	0.67±0.38	0.62±0.63

Ubiquitin Proteasomal System

	ΔΔ/wt	ΔΔbtn2Δ/wt	ΔΔbtn2Δrpn4Δ/wt
TSA2	1.85±0.11	2.47±0.04	0.41±0.06
ERO1	1.65±0.16	2.16±0.45	0.17±0.23
ALD3	1.48±0.79	1.97±0.39	0.82±0.03
CTT1	1.62±0.34	1.66±0.04	2.10±0.19
HSP31	1.17±0.24	1.48±0.22	0.81±0.19
AHP1	1.05±0.33	1.21±0.20	1.41±0.37
GRX2	1.17±0.45	1.19±0.10	-0.15±0.24
TRX2	0.51±0.07	1.11±0.35	-0.40±0.14
PRX1	1.00±0.39	1.09±0.24	-0.06±0.06
GTT1	0.82±0.31	0.87±0.24	-0.13±0.03
MIA40	0.65±0.08	0.81±0.27	0.16±0.03
SOD2	0.55±0.29	0.79±0.12	0.24±0.52
SOD1	0.68±0.35	0.79±0.23	0.21±0.08
HYR1	0.64±0.27	0.75±0.02	0.27±0.03

Oxidative stress response

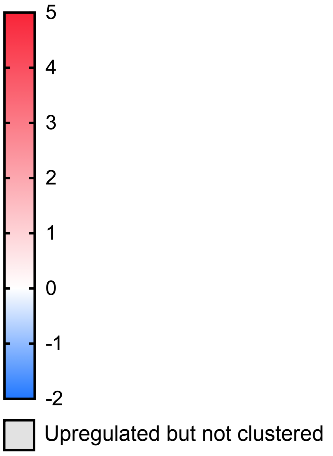

Figure S4

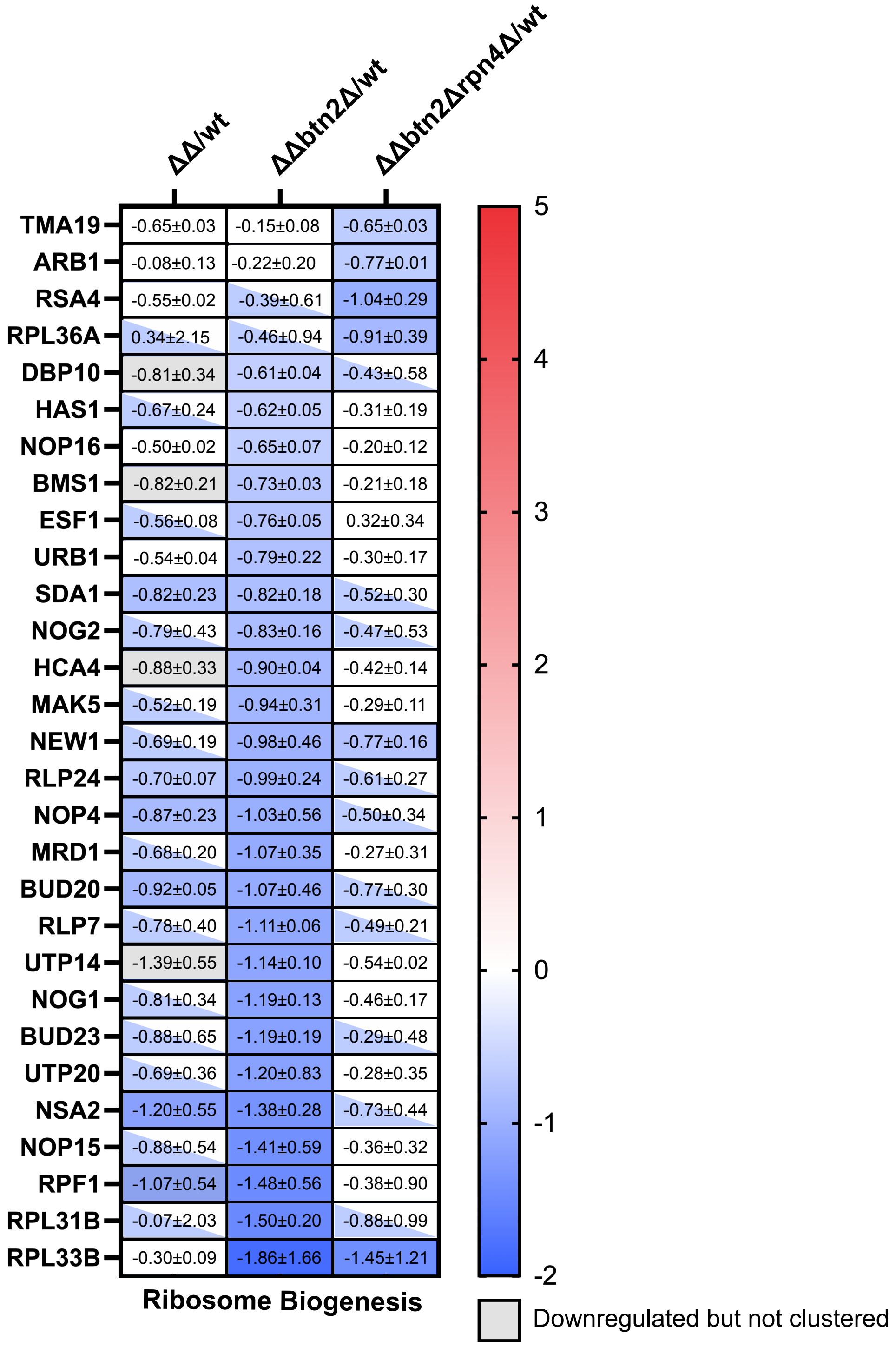
ΔΔ/wt
ΔΔbtn2Δ/wt
ΔΔbtn2Δrpn4Δ/wt
TMA19 -0.65±0.03 -0.15±0.08 -0.65±0.03
ARB1 -0.08±0.13 -0.22±0.20 -0.77±0.01
RSA4 -0.55±0.02 -0.39±0.61 -1.04±0.29
RPL36A 0.34±2.15 -0.46±0.94 -0.91±0.39
DBP10 -0.81±0.34 -0.61±0.04 -0.43±0.58
HAS1 -0.67±0.24 -0.62±0.05 -0.31±0.19
NOP16 -0.50±0.02 -0.65±0.07 -0.20±0.12
BMS1 -0.82±0.21 -0.73±0.03 -0.21±0.18
ESF1 -0.56±0.08 -0.76±0.05 0.32±0.34
URB1 -0.54±0.04 -0.79±0.22 -0.30±0.17
SDA1 -0.82±0.23 -0.82±0.18 -0.52±0.30
NOG2 -0.79±0.43 -0.83±0.16 -0.47±0.53
HCA4 -0.88±0.33 -0.90±0.04 -0.42±0.14
MAK5 -0.52±0.19 -0.94±0.31 -0.29±0.11
NEW1 -0.69±0.19 -0.98±0.46 -0.77±0.16
RLP24 -0.70±0.07 -0.99±0.24 -0.61±0.27
NOP4 -0.87±0.23 -1.03±0.56 -0.50±0.34
MRD1 -0.68±0.20 -1.07±0.35 -0.27±0.31
BUD20 -0.92±0.05 -1.07±0.46 -0.77±0.30
RLP7 -0.78±0.40 -1.11±0.06 -0.49±0.21
UTP14 -1.39±0.55 -1.14±0.10 -0.54±0.02
NOG1 -0.81±0.34 -1.19±0.13 -0.46±0.17
BUD23 -0.88±0.65 -1.19±0.19 -0.29±0.48
UTP20 -0.69±0.36 -1.20±0.83 -0.28±0.35
NSA2 -1.20±0.55 -1.38±0.28 -0.73±0.44
NOP15 -0.88±0.54 -1.41±0.59 -0.36±0.32
RPF1 -1.07±0.54 -1.48±0.56 -0.38±0.90
RPL31B -0.07±2.03 -1.50±0.20 -0.88±0.99
RPL33B -0.30±0.09 -1.86±1.66 -1.45±1.21
Ribosome Biogenesis
5
4
3
2
1
0
-1
-2
Downregulated but not clustered

Figure S5

A

Transcription factor	% in user set	p-value
Hsf1p	96.15%	0.00^{E+00}
Msn4p	100.00%	0.00^{E+00}
Msn2p	100.00%	0.00^{E+00}
Skn7p	69.23%	1.71^{E-10}

Heat Shock Response

Transcription factor	% in user set	p-value
Rpn4p	100.00%	0.00^{E+00}
Pdr3p	100.00%	0.00^{E+00}
Yap1p	100.00%	0.00^{E+00}
Pdr1p	96.77%	5.95^{E-05}

Ubiquitin Proteasomal System

Transcription factor	% in user set	p-value
Msn4p	100.00%	0.00^{E+00}
Yap1p	100.00%	0.00^{E+00}
Aft2p	64.29%	1.57^{E-09}
Cad1p	50.00%	2.84^{E-06}
Msn2p	92.86%	1.69^{E-05}

Oxidative stress response

Transcription factor	% in user set	p-value
Sfp1p	79.31%	5.28^{E-05}

Ribosome Biogenesis

B

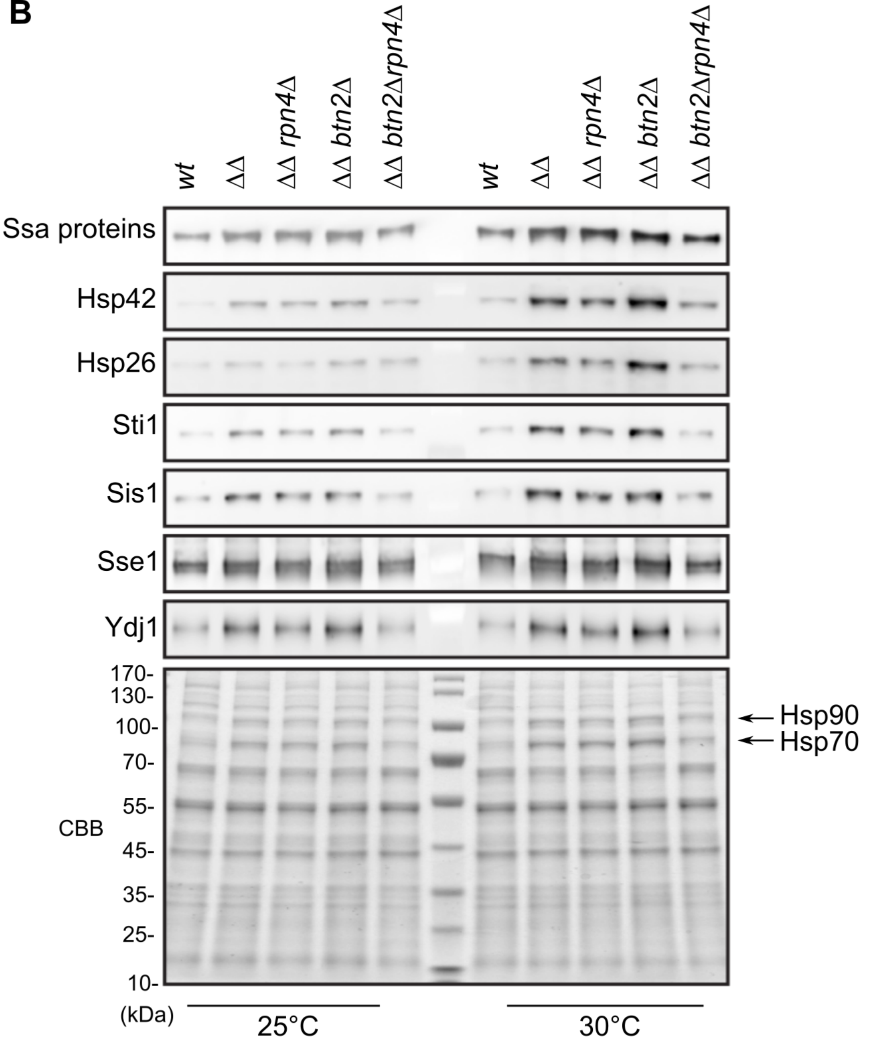

C

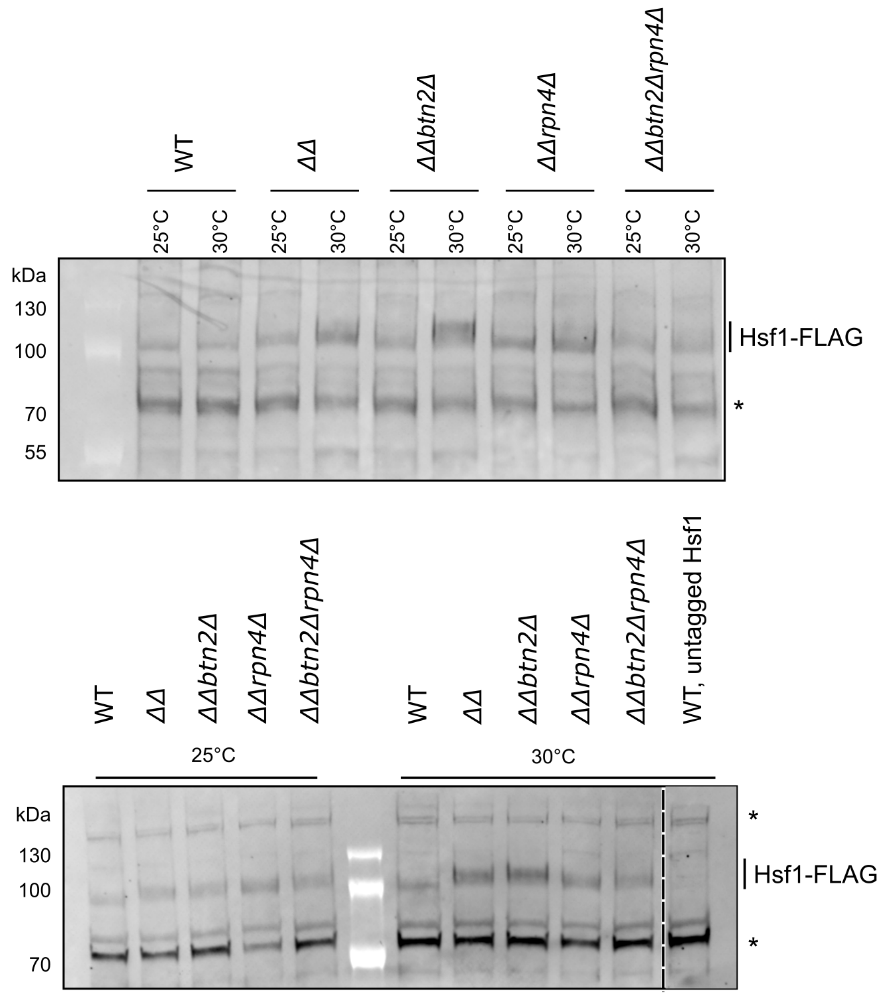

Figure S6

Supplementary Figure Legends

Figure S1

Sequestrase Btn2 and the UPS do not genetically interact. (A/B) Five-fold serial dilutions of indicated *S. cerevisiae* wild type (wt) and mutant cells (*ΔΔ: hsp104Δ fes1Δ*) were spotted on YPD-plates and incubated at indicated temperatures for 2 days. A: YPD plates included 0.002% (v/v) DMSO or MG132 (20 µM) (C) Five-fold serial dilutions of indicated *S. cerevisiae* cells (*ΔΔ: hsp104Δ fes1Δ*) were spotted on SC plates and incubated at indicated temperatures for 3 days. OE: yeast cells harbouring an additional plasmid for increased Rpn4 expression.

Figure S2

Global effects of proteostasis mutants on protein expression. (A/B) Protein levels were compared between *ΔΔ (hsp104Δ fes1Δ*) and wt cells (A) and *ΔΔbtn2Δrpn4Δ* and wt cells (B) at 30°C. For each protein, the x axis show the average $\log_2$ fold change and the y axis shows the p-value for that difference calculated by t-test (two-tailed; n = 2). Proteins belonging to the indicated functional pathways and being significantly up- or downregulated (threshold: 1.5-fold) are highlighted. Proteins lining up on the right border (A) are upregulated at least 5-fold The dashed lines indicate the p-value threshold of 0.05.

Figure S3

Reducing UPS activity in yeast Hsp70 capacity mutants partially reverts cellular stress responses. (A/C-E): Upregulated (> 2-fold) or downregulated (< 2-fold) proteins of indicated strain comparisons, determined by SILAC analysis, were clustered according to a common cellular function. Cluster functions and members and their interconnections are indicated. (B) Sizes of up- and down-regulated clusters, derived from comparisons of indicated mutant cells (*ΔΔ: hsp104Δ fes1Δ*) with WT cells are provided.

Figure S4

Heat map showing upregulation of members of diverse stress response pathways in indicated proteostasis mutant cells. Changes in protein levels are shown as log2-scale. Proteins that did not pass the set threshold (2-fold change) in a specific mutant are shown in white or in mixed colours (white/pink) if the threshold was only passed in one out of two replicates. Proteins that passed the threshold but were not clustered due to too low cluster size (<5) are shown in grey.

Figure S5

Heat map showing downregulation of ribosome biogenesis factors in indicated proteostasis mutant cells. Changes in protein levels are shown as log2-scale. Proteins that did not pass the set threshold (2-fold change) in a specific mutant are shown in white or in mixed colours (white/blue) if the threshold was only passed in one out of two replicates. Proteins that passed the threshold but were not clustered due to too low cluster size (<5) are shown in grey.

Figure S6

Control of stress responses in Hsp70 capacity mutants. (A) Transcription factors regulating the indicated stress response pathways were determined using the YEASTRACT+ database (www.yeastract.com). The relative proportion of genes controlled by the respective transcription factors and respective p-values are provided. (B) *S. cerevisiae* proteostasis mutants were grown at 25°C and 30°C. Equal amounts of total cell lysates were analyzed by Coomassie-stained SDS-PAGE (CBB, bottom). Positions of Hsp70 and Hsp90 chaperones are shown. Levels of indicated chaperones were determined by western blot analysis. (C) Indicated yeast cells (*ΔΔ: hsp104Δ fes1Δ*) expressing functional Hsf1-FLAG were grown at 25°C or 30°C to mid-log growth phase. Total protein extracts were generated and separated by SDS-PAGE and Hsf1 levels were determined by western blot analysis using FLAG-specific antibodies. Phosphorylation of Hsf1 leads to an upshift in the running position. "*" indicates non-specific, cross-reacting bands that serves as loading control.
